# Perceptions and public health risks of the bat-human interface in households from fragmented rural landscapes in southern Chile

**DOI:** 10.1371/journal.pone.0353070

**Published:** 2026-07-06

**Authors:** José Miguel Hernández-Agudelo, Macarena Schenffeldt, Claudio Verdugo, Gerardo Acosta-Jamett, Natalia Castro, Christopher Hamilton-West, Annia Rodríguez-San Pedro, Cristobal Verdugo

**Affiliations:** 1 Instituto de Medicina Preventiva Veterinaria, Facultad de Ciencias Veterinarias, Universidad Austral de Chile, Valdivia, Chile; 2 Facultad de Medicina Veterinaria, Universidad San Sebastián, Puerto Montt, Chile; 3 Instituto de Patología Animal, Facultad de Ciencias Veterinarias, Universidad Austral de Chile, Valdivia, Chile; 4 Center for Surveillance and Evolution of Infectious Diseases, Universidad Austral de Chile, Valdivia, Chile; 5 SENTINET: Surveillance, Epidemiology, and New Technologies for Infectious Emerging Threats, Santiago, Chile; 6 Departamento de Medicina Preventiva Animal, Facultad de Ciencias Veterinarias y Pecuarias, Universidad de Chile, Santiago, Chile; 7 Centro de Investigación e Innovación para el Cambio Climático, Facultad de Ciencias, Universidad Santo Tomás, Santiago, Chile; University of Ibadan Faculty of Veterinary Medicine, NIGERIA

## Abstract

Land-use change and habitat fragmentation in southern Chile have favored synanthropic bat species, promoting their contact with domestic animals and humans, representing a potential risk of exposure. This study aimed to characterize dwellings with bat colonies in rural zones of the Los Ríos and Los Lagos regions, estimate the frequency of human–bat contact, and determine the health risk perception from residents. A cross-sectional descriptive study was conducted in 69 rural dwellings. A structured survey was used to gather information on demographics, household characteristics, frequency of contacts, and risk perception. Data regarding contact levels and household attributes were evaluated using descriptive statistics and non-parametric tests. Surveyed dwellings were predominantly wooden construction, whit 67% presenting unsealed openings, and bat presence was reported in 46% of households. Higher contact levels were significantly associated with the presence of pets (dogs p = 0.0014, V = 0.69; cats p = 0.021, V = 0.5) and vulnerable residents (minors or seniors, p = 0.0014, V = 0.67). Conversely, dwelling structure did not present a clear pattern differentiating the contact level. Although 84% of respondents acknowledged disease transmission risk, primarily rabies, there was a significant gap in risk perception regarding livestock, in addition to a lack of consistent preventive actions regardless of health risk knowledge. This perception paradox requires a One Health educational approach, beyond traditional rabies management. These results highlight a high frequency of cohabitation creating significant potential for exposure and representing a potential public health concern in southern Chile.

## Introduction

Emerging infectious diseases remain a major global threat, with substantial consequences for public health and the economy [1,2]. Most emerging human pathogens are zoonotic in origin [3,4], and many emergence events are associated with wildlife reservoirs and anthropogenic environmental change [4,5]. Among the most important drivers are land-use change and habitat fragmentation driven by agricultural expansion, urbanization, and other forms of ecosystem disturbance, all of which can restructure ecological communities, alter host movement and resource use, and increase contact among wildlife, domestic animals, and people [6–8]. These processes create novel epidemiological interfaces that facilitate pathogen transmission, cross-species exposure, and host shifts [9]. Within this context, bats (Order Chiroptera) are a key taxon of interest because they are associated with a wide diversity of microorganisms, including pathogens of major public health relevance [10]. Bats have been globally implicated in the ecology of lyssaviruses, including rabies virus and related viruses [11], and linked to the evolutionary history and emergence of several coronaviruses, including SARS-related coronaviruses, as well as henipaviruses in some regions [12]. Although only a subset of bat-associated microorganisms poses a direct zoonotic risk, bats are especially relevant because many species exhibit high ecological flexibility, persist in human-modified environments, show high mobility, and exploit artificial structures and buildings for roosts when natural refuges are reduced or disturbed [13–16]. As a result, habitat fragmentation can restructure patterns of roost use, foraging, and direct or indirect interspecific contact in ways that are epidemiologically relevant [17,18].

The bat-human interface is therefore a global concern, not only in highly-urbanized areas but particularly in rural and peri-domestic landscapes where people, pets, livestock, and wildlife share space at fine spatial scale [18,19]. Human exposure may occur through the direct handling of bats, bites or scratches, contamination of inhabited spaces with feces or urine, or indirect interactions mediated by domestic animals [18]. Understanding these interactions is crucial for exposure risk management, as the probability of pathogen spillover is influenced not only by ecological proximity, but also by human behavior, knowledge, and perceptions [17,20,21]. Studies have shown that frequent cohabitation with bats often coincides with limited public knowledge of bat-associated diseases, frequently leading to underestimation of the actual risks involved [11,17]. This pattern may reflect normalization of contact through repeated exposure, inconsistent preventive behavior, or other factors influencing risk assessment [17]. Thus, this disconnect between actual exposure pathways and residents’ perceived risk in households cohabiting with bats represents a critical component in understanding zoonotic risk, although it remains an insufficiently characterized dimension of spillover prevention.

In southern Chile, these dynamics are especially relevant. The temperate landscapes of the Los Ríos and Los Lagos regions have undergone extensive fragmentation, as a consequence of deforestation and land-use change associated primarily with agriculture, forestry, livestock grazing, and urbanization [22,23], producing a heterogeneous mosaic of forest remnants, farms, and rural dwellings. In this context, synanthropic bat species such as *Myotis chiloensis* and *Tadarida brasiliensis* frequently use different types of human-made structures as shelters [15,16], increasing the potential for repeated contact with humans and domestic animals. Importantly, these species have been associated with zoonotic agents, including bacteria such as *Bartonella* spp. and *Coxiella burnetii* [24], as well as rabies virus variants [25,26]. This cohabitation is particularly relevant given the demographic profile of the study area; approximately 30% of the population in the Los Ríos and Los Lagos regions lives in areas classified as rural zones, representing the highest rate of rurality in the country [27]. These rural settings are characterized by socioeconomic heterogeneity, limited access to services, and restricted public health information [28], shaping the patterns of exposure and sanitary risk perception. Despite these conditions, the characteristics and frequency of bat-associated household colonization, the intensity of human-bat-domestic animal contact, and the extent to which local residents perceive these interactions as a health risk in rural southern Chile remain largely unquantified.

In this study, we characterized the bat-human interface in rural dwellings associated with colonies in southern Chile. Specifically, we aimed to describe household and structural characteristics associated with bat presence, estimate the frequency of contact among bats, humans, and domestic animals, and assess residents’ perceptions of sanitary risks associated with bat cohabitation. By integrating ecological context with household-level exposure and risk perceptions, this study provides baseline evidence to inform surveillance, risk communication, and preventive strategies in fragmented rural landscapes.

## Materials and methods

### Study area, study design, and participant recruitment

The study was conducted in the Los Ríos and Los Lagos regions of southern Chile, within the Valdivian temperate rainforest ecoregion. Although this area was originally dominated by native temperate forest, it is now characterized by a heterogeneous rural matrix shaped by long-term anthropogenic activities, including agriculture, livestock and commercial forestry plantations, and expanding human settlements [23]. This landscape configuration generates a mosaic of native forest remnants, farms, and dispersed rural dwellings. We conducted a cross-sectional household survey targeting residents of rural dwellings. The recruitment and data collection period spanned from October 10, 2023, to March 29, 2024. Household selection was based on 100 sampling points initially selected for a related project on pathogen emergence. These points were randomly distributed across both regions with a minimum geographic distance of 10 km between points. The selection aimed to ensure broad spatial coverage of different agroecological zones across the region. Final site selection was adjusted according to field accessibility.

At each sampling point, all occupied rural dwellings and associated structures located within the fenced perimeter of the selected property were visited. Given the characteristics of rural settlements in southern Chile, a single property often contains multiple buildings, such as secondary houses for extended family members, sheds, or small community structures (e.g., rural churches). Therefore, no specific threshold distance or maximum number of targeted structures was set per point; instead, all eligible structures within the property boundaries were considered, and adult residents were approached to participate. Eligible participants were required to be at least 18 years old, to have permanent residence in the household area to ensure reliable knowledge of dwelling conditions, and to provide informed consent. Data quality criteria were applied prior to analysis. Surveys were excluded if fewer than 75% of questions had been answered or if critical missing information could not be resolved after three follow-up contact attempts with the respondent.

### Survey instrument

Data was collected using a structured survey applied in-person to adult household residents, in Spanish, and designed and refined after a pilot pre-testing step with input from national experts in veterinary medicine and public health. The final questionnaire consisted of 27 items including both open- and closed-ended questions and was organized into three thematic sections (S1 Table): i) household demographics characterization: number of residents, presence of potentially vulnerable age groups (<18 years and ≥65 years), presence of pets and livestock, livestock management practices, and structural features of the dwelling or animal-housing areas that could facilitate bat entry (e.g., holes, gaps, fissures in walls or roofs); ii) dwelling characteristics and bat presence: direct or indirect evidence of bat presence inside the dwelling or in structures used for animals (bat feces), observed contact between bats and domestic animals, construction materials, estimated age of the dwelling, use of the colonized structure, distance from the main dwelling, duration of known bat presence, control measures adopted, and bat occurrence in surrounding areas; iii) risk perception: awareness of diseases transmitted to humans, pets, and livestock, knowledge of specific diseases, prior reporting to health authorities, rabies vaccination status, and the actions in the event of finding a bat indoor. The respondent contact details and the date of application were recorded for quality-control purposes. During the interview, and with the residents’ consent, photographic records of the dwelling and associated structures were also obtained to document structural characteristics and direct signs of bat presence.

### Statistical analyses

Unanswered questions were coded as missing data, and analyses were conducted using available-case data for each variable. To support descriptive and exploratory comparative analyses, several derived variables were constructed from the questionnaire responses.

First, an ordinal human-bat contact level (contact level) variable was generated to summarize the intensity of exposure at the household level. This variable integrated reported direct sightings of bats, indirect evidence of bat presence (e.g., guano), the location of bat occurrence relative to the household, and reported direct interactions with bats. Contact level was classified into three levels to reflect a gradient of epidemiological exposure risk: (1) low, corresponding to external presence or indirect environmental evidence only (e.g., bats seen flying near the property or guano restricted to exterior walls and unoccupied barns); (2) medium, corresponding to internal presence within the dwelling implying potential direct exposure (e.g., guano or bats found inside the house, but without reported physical contact by humans or pets); and (3) high, corresponding to reported direct physical interaction (e.g., handling, bites, or scratches), observed physical contact between bats and domestic animals, or high frequency access to bats. In this study, “high frequency access” does not refer to a calculated numerical rate, but rather to scenarios where spatial overlap makes recurrent encounters highly probable or unavoidable, such as colonies roosting directly inside high-traffic living areas (e.g., bedrooms or kitchens) or recurrent handling of bats by residents for exclusion purposes. The specific questionnaire responses and scenarios required to classify households into each epidemiological risk gradient are detailed in S2 Table.

Second, a health-risk knowledge score (knowledge score) was calculated as a simple descriptive indicator using an additive ordinal scale from 0 to 3. Respondents received one point for each affirmative response regarding awareness of potential bat disease transmission to: (i) humans, (ii) pets, and (iii) livestock. Therefore, a score of 3 indicated recognition of transmission risks across all three interfaces.

Third, self-reported immediate responses to the presence of a bat indoors were recoded into a mutually-exclusive action category variable into three groups based on their primary reaction: negative/aggressive, including lethal or harmful actions toward the bat; neutral/evasive, including passive responses or non-intrusive removal; and precautionary/informative, including safe containment, precautionary actions during the encounter to avoid physical contact, or seeking assistance from health or veterinary authorities. In addition, binary indicator variables were created for the presence or absence of selected domestic animals (cats, dogs, and bovines), for the presence of vulnerable household members (<18 or ≥65 years), and for structural vulnerability of the dwelling defined a priori as the presence of unsealed holes or gaps in the dwelling, and construction older than 20 years.

The analytical approach was descriptive with exploratory bivariate comparisons. Categorical variables were summarized as frequencies and percentages, and numerical variables as measures of central tendency and dispersion. Proportions were accompanied by 95% confidence intervals when appropriate. Sampling point coordinates were plotted to illustrate the spatial distribution of surveyed households across the Los Ríos and Los Lagos regions. Exploratory associations between human-bat contact level and household characteristics, including the presence of pets, vulnerable household members, and structural vulnerability are strictly exploratory and intended for hypothesis generation. These associations were evaluated using Fisher’s exact test or chi-square tests with simulated p-values based on Monte Carlo resampling simulation (B = 5000 iterations) accounting for small cell counts. To assess the magnitude of these exploratory associations, effect-size estimates (Cramer’s V) with 95% confidence intervals (CI) were calculated and presented alongside complete contingency tables. Spearman’s rank correlation was used to assess the relationship between the reported duration of bat colony presence and risk-perception variables. Statistical significance was established at p ≤ 0.05. All statistical analyses were performed using R software, version 4.4.1 (R Core Team, 2024).

### Ethics statement

The data collection for this study was conducted as part of a larger ecological research project (ANILLO ATE220062) focused on bat and wild rodent sampling. Accordingly, the overarching study protocol was reviewed and approved by the Institutional Committee for the Care and Use of Animals in Research (CICUA) of the Universidad Austral de Chile (protocol code 510/2023). The household questionnaire and the informed consent procedures for human participants were submitted as a formal amendment to this protocol and were specifically reviewed and approved by the aforementioned committee, giventhat some of the animal traps were placed within the interviewees’ properties. All procedures involving human participants were performed in accordance with the Declaration of Helsinki and relevant national guidelines. Written informed consent was obtained from all individual participants before commencing the survey. Only adult residents (18 years or older) were recruited for the surveys; therefore, no consent from parents or guardians was required as no minors were primary participants in the survey. The authors had access to information that could identify individual participants during the data collection phase (e.g., names and phone numbers for logistical coordination); however, all data were fully anonymized before the analysis phase to ensure participant confidentiality. No identifying information is present in the final dataset.

## Results

### Household sample and baseline characteristics

**Fig 1:**
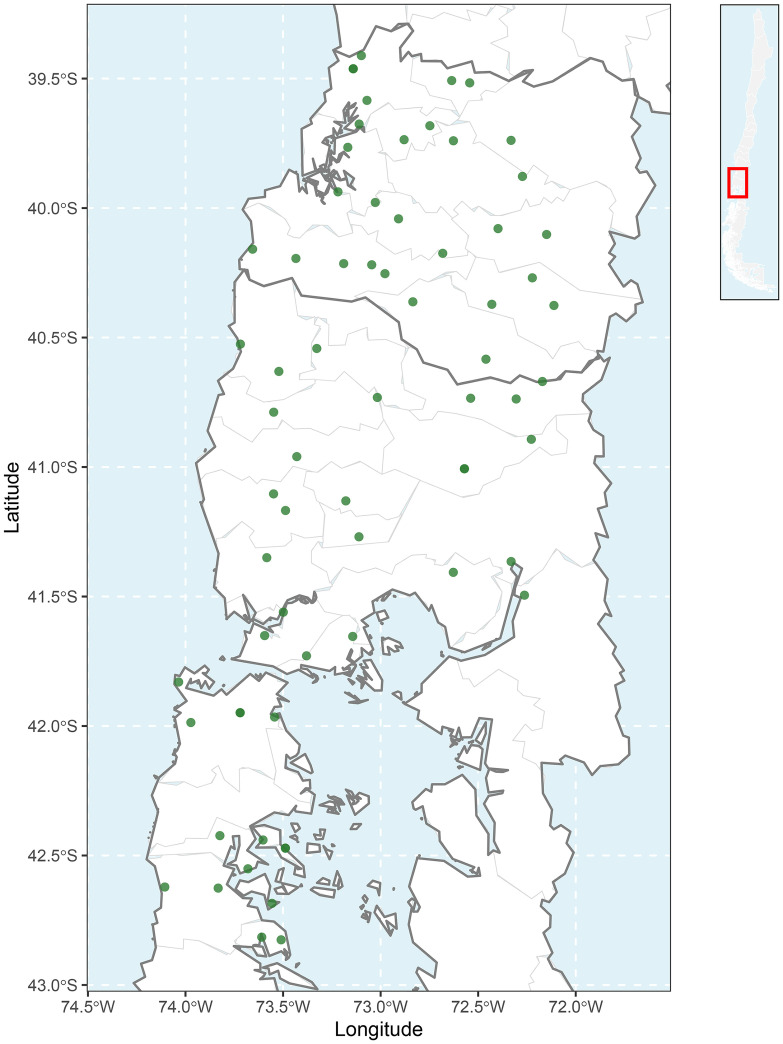
Spatial distribution of the surveyed households in southern Chile. The main map displays the geographic location of the 69 sampling points across the Los Ríos and Los Lagos regions. The inset map (top right) highlights the study area (red box).

A total of 72 surveys were initially obtained from rural households in southern Chile. Of these, 3 surveys did not meet the data quality criteria (e.g., fewer than 75% of questions answered) and were therefore excluded. Thus, 69 valid surveys were included in the final analysis, comprising 32 households from the Los Ríos region (46.4%) and 37 from the Los Lagos region (53.6%). Surveyed households were distributed across a diverse range of agroecological zones within the study area (Fig 1).

Of the 69 dwellings surveyed, 47 (68%) were reported as being regularly inhabited overnight. Across these inhabited households, 138 residents were recorded, with a mean household size of 2.9 persons, a median of 3, and an interquartile range of 2–4. Among all recorded residents, 59/138 (42.8%) belonged to potentially vulnerable age groups, including 32 individuals younger than 18 years and 27 who were 65 years or older (Table 1). Pets were reported in 49 of the 69 households surveyed (71.0%). When distinguishing by specific pet ownership, 31 of the total households (44.9%) owned both dogs and cats, 14 (20.3%) owned only dogs, and 4 (5.8%) owned only cats. Livestock was reported in 41/69 dwellings (59.4%). Bovines were the most frequently reported (27 dwellings), followed by poultry (26 dwellings) and sheep (17 dwellings). Other species, including horses, pigs, goats, llamas, and bees, were each reported in fewer than 12 households (Table 1).

**Table 1:** Demographic, household, and agricultural characteristics of surveyed rural dwellings in southern Chile.

Characteristic	Frequency (n)	Percentage (%)
**Study Region (N = 69 households)**		
Los Lagos	37	53.6
Los Ríos	32	46.4
**Household Occupancy**		
Regularly inhabited overnight (out of 69)	47	68.1
Total recorded residents	138	–
Household size, number of persons (Mean / Median [IQR])	2.9 / 3 [2–4]	–
**Resident Age Distribution (N = 138 individuals)**		
Potentially vulnerable age groups	59	42.8
Minors (<18 years)	32	23.2
Seniors (≥65 years)	27	19.6
**Presence of Domestic Animals (N = 69 households)**		
**Households with pets**	49	71
Both dogs and cats	31	44.9
Only dogs	14	20.3
Only cats	4	5.8
**Households with livestock**	41	59.4
Bovines	27	39.1
Poultry	26	37.7
Sheep	17	24.6

### Bat occurrence and roost/structural characteristics

Evidence of bat presence, based on direct sightings or indirect signs, was reported in 32 of 69 households surveyed (46.4%). In addition, 46/69of respondents (67%) acknowledged unsealed openings, such as holes, gaps, or fissures, that could facilitate bat entry into the dwelling. Among the 47 dwellings reported as regularly inhabited overnight, 27 (57.4%) had observed bat feces inside living spaces. Evidence of bat feces was less frequently reported in areas used by domestic animals, including 5/69 (7.2%) in pet sleeping areas and 3/69 (4.3%) in livestock areas. Observed direct contact between bats and domestic animals was uncommon: 8 of 49 pet-owning households (16.3%) affirmed witnessing contact between their pets and bats, whereas no respondents observed direct bat-livestock contact (Table 2).

**Table 2:** Household-level indicators of bat presence and potential exposure.

Indicator	Total Evaluated (N)	Frequency (n)	Percentage (%)	95% CI (%)
Reported bat presence (sightings and/or signs)	69	32	46.4	34.3–58.8
Structural vulnerability (presence of unsealed openings, > 20 years)	69	46	66.7	54.3–77.6
Bat feces observed inside primary dwelling	47	27	57.4	44.2–71.7
Bat feces observed in pet sleeping areas	49	5	10.2	3.4-22.2
Bat feces observed in livestock areas	41	3	7.3	1.5–19.9
Observed bat-pet contact	49	8	16.3	7.3-29.7

Regarding the intensity of human-bat interactions, the 31 households with confirmed bat presence and interactions were classified into low (11/31, 35.5%), medium (11/31, 35.5%), and high (9/31, 29.0%) contact levels. Most reported colonies were in the primary residence (19/31, 61.3%), followed by community buildings (9/31, 29%,), and abandoned or unused structures (3/31, 9.7%). All 31 colonized structures were reported as wooden, often combined with zinc roofing (56%). Respondents reported bat colonies across a broad range of building ages, although the most frequently reported age category was 21–40 years (8/31, 25.8%). However, colonies were also present in newer constructions (<20 years, 12.5%), as well as in older structures (>80 years, 9.4%). Reported duration of known bat presence was variable, with the most frequent category (31%) reporting 6–10 years.

**Fig 2:**
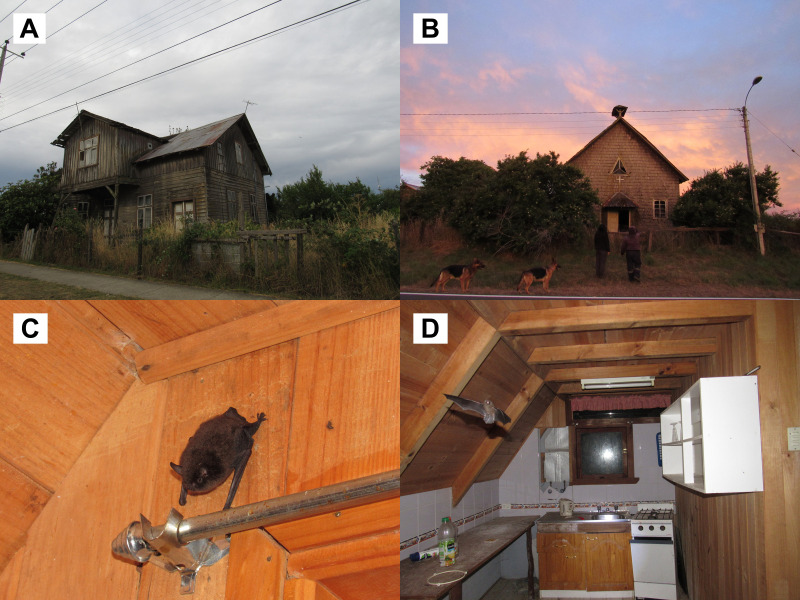
Photographic records of dwellings and bat presence in southern Chile. **(A)** Two-story wooden rural dwelling with zinc roofing, showing structural deterioration and unsealed openings facilitating bat entry. **(B)** A wooden community building (rural parish) acting as a bat roost. **(C)** An individual bat, identified as *Myotis chiloensis*, roosting on an interior wooden wall. **(D)** An individual *M. chiloensis* captured in flight inside the kitchen of an inhabited household.

Among all households reporting bat presence (n = 31), 17/31(54.8%) indicated that some form of control or exclusion measure had been attempted. Photographic records and visual inspection further supported the survey-based characterization of the structural conditions and the synanthropic behavior of bats, corroborating the frequent colonization of deteriorated wooden dwellings and community buildings with potential access points, as well as bats roosting on interior wooden walls and actively flying within functional rooms of the houses (Fig 2).

### Bat-human contact and associated household factors

To evaluate the factors associated with exposure intensity, exploratory bivariate associations were performed on the sub-sample of households reporting bat presence and sufficient interaction data for classification (n = 31). Bat-human contact intensity varied significantly based on household composition and the presence of companion animals. High contact levels were strongly associated with the presence of pets. Specifically, the presence of dogs showed a large effect size and a significant association with higher contact levels (p = 0.0014, Cramer’s V = 0.69, 95% CI: 0.31–1.00); in households with dogs, 16/17 (94.1%) were classified as medium or high contact, compared to 4/14 (28.6%) in households without dogs. Similarly, households with cats showed a higher proportion of high contact (46.2%) than those without cats (16.7%; p = 0.0210, Cramer’s V = 0.50, 95% CI: 0.07–0.83).

The presence of potentially vulnerable household members (minors or seniors) was also significantly associated with higher contact intensity (p = 0.0014, Cramer’s V = 0.67, 95% CI: 0.29–1.00). In this group, 100% of households (14/14) were classified as medium or high contact, whereas only 6/17 (35.3%) of non-vulnerable households reached these levels. In contrast, structural vulnerability (p = 0.2947, Cramer’s V = 0.33, 95% CI: 0.00–0.65) and the presence of livestock such as bovines (p = 0.3413, Cramer’s V = 0.28, 95% CI: 0.00–0.60) did not present statistically significant associations with contact intensity level. Complete contingency tables for these associations, detailing the cross-tabulated frequencies, effect-size estimates (Cramer’s V), and 95% CIs, are provided in S3-S7 Tables in S1 File. Overall, these results suggest that once bats are present in the household environment, contact intensity is more strongly related to household composition and pet presence than to structural deficiencies alone.

### Knowledge and perceived risk of bat-associated diseases

Most interviewees recognized awareness that bats could transmit diseases to humans (58/69, 84%, 95% CI: 73–91%). Among those respondents, 51/58 (88%, 95% CI: 77–94%) specifically identified rabies, whereas the remaining 7/58 (12%, 95% CI: 6–24%) were unable to name any disease suggesting a limited knowledge of specific diseases. Further, perceived transmission risk to domestic animals was substantially lower, only 35/69 interviewees (51%, 95% CI: 39–63%) recognized a potential risk to pets, and only 22/69 interviewees (32%, 95% CI: 21–44%) perceived a risk to livestock.

The accumulated health-risk knowledge score was calculated for a subset of respondents (n = 31), representing those from bat-colonized households who provided complete responses regarding disease transmission risk across all three evaluated interfaces. Within this group, 4/31 (12.9%) scored 0, indicating no awareness of bat-associated disease risk across any of the interfaces, whereas 9/31 (29.0%) scored 3, reflecting recognition of potential transmission to humans, pets, and livestock. Intermediate scores of 1 and 2 were recorded in 10/31 (32.3%) and 8/31 (25.8%) respondents, respectively.

Intended actions following the presence of a bat indoors varied widely, regardless of the respondent’s awareness. Among the 18 respondents with coded open-ended answers, 5/18 (27.8%) described negative or aggressive actions, 6/18 (33.3%) described neutral or evasive measures, and 7/18 (38.9%) described precautionary or informative actions. Similarly, awareness of rabies was not statistically associated with reported human-bat contact level (p = 0.155), suggesting that general awareness of disease risk did not translate into a uniform preventive response.

Regarding the preventive measures and health history, when asked if they or any family member were vaccinated against rabies, only 9/69 (13.0%) responded affirmatively, whereas the vast majority (55/69, 79.7%) reported not being vaccinated. A small proportion either did not know (3/69, 4.3%) or did not answer the question (2/69, 2.9%).

## Discussion

**Fig 3:**
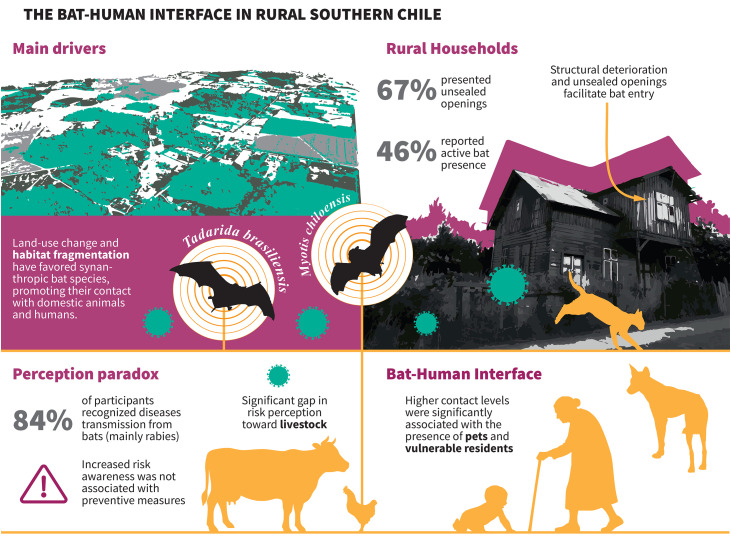
Overview of the main findings of interactions between bats, humans, pets, and livestock in fragmented landscape in southern Chile. The diagram illustrates the multiple contact pathways among synanthropic bats, domestic animal (pets and livestock), and vulnerable human groups (infants and seniors) within the rural domestic interface. The dog silhouette is reprinted from PhyloPic (*Canis familiaris dingo* icon by Sam Fraser-Smith and T. Michael Keesey) under a CC BY license. All other silhouettes (cat, human figures, cow, and chicken) are in the public domain (CC0 1.0).

This study reveals frequent human-bat cohabitation in rural households of southern Chile highlighting the relevance of this interface in fragmented landscapes. Bat presence was reported in nearly half of the households surveyed, signs of bat activity within inhabited spaces were common, and more than half of bat-positive households had already attempted some form of control or exclusion. These findings indicate that contact between bats and humans in this system is not sporadic, but rather a recurrent feature of rural domestic environments (Fig 3).

This pattern is ecologically plausible in the context of the extensive habitat fragmentation of southern Chile [22,29] characterized by a landscape where human, domestic animals, and wildlife habitats overlap. Under these conditions, human-made structures, such as older wooden constructions, offer an accessible and stable roosting alternative [14,19], particularly where natural refuges have been reduced or disturbed by deforestation. Six bat species have been described in Los Ríos and Los Lagos regions: *Tadarida brasiliensis*, *Myotis chiloensis*, *Histiotus montanus*, *Histiotus magellanicus*, *Lasiurus varius*, and *Lasiurus villosissimus* [16]. They are all insectivorous and naturally inhabit native forests, commonly roosting in tree hollows. However, *Tadarida brasiliensis* and *Myotis chiloensis* exhibit synanthropic behaviors, frequently colonizing anthropogenic structures such as attics and wall cavities in both rural and urban areas. This aligns with the behavior observed in other bat species globally, where adaptation to live in close association with humans occurs, particularly in human-modified landscapes [14,30]. The predominance of structural deterioration and unsealed openings likely facilitating bat entry further supports that building characteristics and maintenance are key determinants enabling colonization by common bat species [15,16]. In addition, the visual confirmation of bats documented roosting and flying inside inhabited rooms of these dwellings underscores that this synanthropic use of spaces in this system is not limited to peripheral structures but extended to core domestic environment.

The assessment highlights the importance of wildlife, domestic animals, and human interactions in rural settings. Higher contact levels were significantly reported in households with pets, particularly dogs (p = 0.0014, Cramer’s V = 0.69) and cats (p = 0.021, Cramer’s V = 0.50). Although direct bat-pet interactions were occasionally reported (16%), these events are likely underestimated given the nocturnal behavior of bats and cryptic interactions [17,31]. The observed frequencies and strong magnitude of these effects (V > 0.50) suggest companion animals substantially increase the likelihood of higher exposure encounters by several non-exclusive interactions, such as bringing bats into homes [32–34], interacting with injured or dead bats, and bringing bats into closer proximity with people [11]. From a One Health perspective, pets can act as sentinels of bat presence, drawing human attention and serving as recognized intermediary hosts for zoonotic pathogens, including lyssaviruses [32,33,35], thereby linking wildlife and humans. The frequent coexistence of synanthropic bats, pets, and livestock (present in 59% of households) creates multi-host contact pathways with potential implications for pathogen spillovers.

Similarly important, there was a significant association between higher contact intensity and households with minors or older adults (p = 0.0014, Cramer’s V = 0.67). Although this association requires further investigation given the limitations in sample size, different activity patterns may suggest that household dynamics can influence the likelihood of exposure [11,30]. In children, increased curiosity or lower perception of danger could increase the likelihood of approaching or handling bats. In older adults, chronic cohabitation with deteriorated structures, reduced capacity to maintain or repair housing facilities or to implement exclusion measures, or reduced access to preventive information may contribute to prolonged and frequent exposure. In contrast, although structural openings were highly prevalent which supports their role in facilitating bat entry, they were not significantly associated with higher levels of human-bat contact. This suggests that structural integrity may be crucial for preventing access and colonization [14,19], but once bats are established, other factors like household composition and pet presence are more relevant in the intensity of subsequent contact with people. Approximately 30% of the Los Ríos and Los Lagos regions live in areas classified as rural zones, representing the highest rate of rurality in the country [27], characterized by an advanced demographic transition due to a progressive aging of the population [36]. Thus, rural households in southern Chile, like those in many other countries in the Southern Hemisphere, face multidimensional demographic, socioeconomic, and structural challenges. These compounding factors perpetuate conditions that increase the risk of bat exposure, highlighting the urgent need for context-adapted prevention and risk communication strategies. A marked disconnect between perceived risk and specific, actionable knowledge, a phenomenon previously reported for other contexts [20,21], was observed. Although most respondents recognized that bats could transmit diseases to humans, this awareness was largely restricted to rabies. Overlooking other relevant pathogens potentially associated with Chilean bats, such as *Bartonella* spp. and *Coxiella burnetii* [24], highlights a significant public health gap. Similarly, long-term cohabitation with bats does not necessarily seem to promote greater knowledge or predictably alter interaction levels, potentially reflecting habituation or risk normalization [17]. This “perception paradox” leads residents to recognize a general sanitary concern—which does not necessarily translate into consistent protective behaviors—and to underestimate the diversity of epidemiologically relevant risks associated with cohabitation [37] and the broader animal health dimensions of the bat-human interface.

The particularly low perception of risk to livestock suggests a latent vulnerability for the local livestock industry, likely attributed to the absence of the common vampire (*Desmodus rotundus*) in these southern latitudes, which are instead dominated by insectivorous bats. Thus, rural residents from southern Chile may be less likely to perceive bats with animal-health threats, particularly in production systems. However, this gap remains critical given that these regions concentrate the large proportion of the national cattle herd, accounting for approximately 82% of Chile’s total milk production [38]. Bat-borne rabies involving spillovers to livestock is primarily documented as a veterinarian issue, where surveillance data confirms bats as the primary reservoir maintaining viral variants that spill over into productive herds through accidental encounters [25,26].

Awareness of rabies did not correspond to a consistent pattern of intended response action when bats were encountered indoors. Reported actions ranged from aggressive removal to passive avoidance and precautionary responses, suggesting that general awareness of disease risk does not translate into appropriate protective behavior. This discrepancy highlights a clear lack of consistent, informed protocols for managing bat encounters in these communities. This disconnect mirrors findings even for high-income countries, where risky behaviors such as intentional handling (often to “help” a bat), or delays seeking appropriate care following exposure, are reported [11,33,35]. In this line, repairing structural deficiencies, such as cracks and gaps, which are the initial source of bat entry into dwellings in Chile [16], seems highly unlikely if residents do not perceive a severe or tangible threat [33]. This could be reinforced by the low perception of the actual risk of rabies transmission to humans in the country, despite eradication policies [16].

The survey revealed that only 13% of households reported having a member vaccinated against rabies. In Chile, human rabies vaccination is not applied as a preventive population-wide measure, but rather strictly as Post-Exposure Prophylaxis (PEP) following an animal bite or direct bat exposure, as per the national guidelines [39]. Therefore, 13% likely reflects the historical incidence of animal bites or wildlife exposures requiring PEP among these families rather than programmatic coverage. For regional context, the demand for PEP in these areas is notable; recent data from 2023 indicates that 2,453 and 4,702 first doses of the antirabies vaccine were administered in the Los Ríos and Los Lagos regions, respectively [40]. Despite this regional demand for PEP, the low proportion of vaccinated individuals in the sample combined with the frequent reports of human-bat cohabitation underscores a potential vulnerability in these rural communities if exposures go unrecognized or unreported. In this line, repairing structural deficiencies, such as cracks and gaps, which are the initial source of bat entry into dwellings in Chile, seems highly unlikely if residents do not perceive a severe or tangible threat.

This lack of risk perception is reinforced by the extremely low incidence of human rabies in the country. Chile has not recorded a human rabies case from a canine variant since 1972, and only two isolated human cases have been reported nationally in recent decades (in 1996 and 2013), neither of which occurred in the Los Ríos or Los Lagos regions [40]. Consequently, the actual clinical rabies rate in the studied populations is zero. The absence of recent human cases creates an epidemiological paradox. The exposure hazard remains highly tangible due to the sustained endemicity of the virus in synanthropic bats; longitudinal surveillance data from the Chilean Public Health Institute (ISP) reveals that nationwide rabies positivity among tested bats has consistently increased from 3.9% in the 2008–2013 period to range between 7% and 10% over the last decade, reaching 8.1% in 2023 and 7.9% in 2024 [41–44]. Furthermore, the canine rabies variant has been eradicated in Chile since 1990, meaning that 100% of recent animal rabies cases including sporadic spillovers to domestic dogs and cats originate strictly from bat variants, predominantly maintained by *Tadarida brasiliensis* [42–44]. Despite this continuous ecological hazard and their frequent colonization of rural households, the lack of visible human cases fosters a false sense of security and a remarkably low perception of the actual risk among residents [26].

Several limitations should be considered when interpreting these findings. First, the data relied on self-reported information and is therefore subject to potential recall and social desirability biases [17], particularly for sensitive behaviors such as handling or intentional killing bats. Moreover, reported rates of bat-animal interactions were likely underestimated because such interactions are often cryptic, brief, and often nocturnal [17,31]. Furthermore, the health-risk knowledge score utilized in the analysis serves strictly as a basic descriptive indicator of general awareness regarding potential disease transmission from bats to humans, pets, and livestock. It does not constitute a validated psychometric measure, nor does it capture the depth, accuracy, or practical applicability of the residents’ sanitary knowledge. Second, the sample was not probabilistic, which limits formal generalization of the findings to all rural households in southern Chile. However, despite the sample size and the accessibility-based selection of households near predefined ecological points, the sampling design was spatially random, covering a broad geographic coverage across both study regions. This extensive territorial reach constitutes a major strength of the study, supporting a robust regional characterization of the bat-human interface across the surveyed rural landscape in southern Chile. Finally, while our study frames these interactions within the broader context of public health, we did not directly measure pathogen presence in the bats, confirm specific bite or scratch events leading to clinical disease, or quantify actual infection risk. Therefore, our findings should be interpreted as evidence of potential exposure pathways, risk-related household conditions, and perceived risk among residents, which together characterize the potential for zoonotic spillover rather than direct pathogen transmission.

## Conclusion

In conclusion, rural households in southern Chile represent a frequent interface among synanthropic bats, humans, pets, and livestock, creating a high potential for zoonotic exposure. Bat cohabitation was common and was often facilitated by structurally permissive housing conditions, particularly those associated with older buildings and certain construction materials. Higher contact intensity was more likely in households with companion animals and in those including potentially vulnerable age groups. Yet general awareness of bat-associated disease risks did not translate into broad recognition of animal health implications or into consistent preventive responses. These findings reveal a critical gap in the management of bat-associated exposure in rural environments and support the need for One Health-based surveillance, risk communication, and household-level prevention strategies that address both pathogen awareness and the practical management of bat encounters in neglected rural communities.

## Supporting information

S1 Table Survey for the characterization of bat presence and sanitary risk perception. English-translated version of the questionnaire administered to rural households.
(DOCX)

S2 Table Coding matrix for the ordinal “human-bat contact level” variable based on questionnaire responses. (DOCX)

S1 File S3-S7 Tables. Contingency tables for bivariate associations between household characteristics and human-bat contact levels.
(DOCX)
