# Supplementary material for: Perceptions and public health risks of the bat-human interface in households from fragmented rural landscapes in southern Chile
Source: PLoS One. 2026 Jul 6;21(7):e0353070. doi: 10.1371/journal.pone.0353070 (PMC13336185; doi:10.1371/journal.pone.0353070)
Supplement: S1 Table — English-translated version of the questionnaire administered to rural households. (DOCX) [file pone.0353070.s001.docx]

**S1 Table: SURVEY FOR THE CHARACTERIZATION OF BAT PRESENCE AND SANITARY RISK PERCEPTION IN SOUTHERN CHILE**

**ANILLO PROJECT ATE220062**

**Date: _________________________        ID: ___________________________**

**Full Name: _________________________________________________**

**Contact Phone: _____________________________________________**

| **N°** | **Question** | **Response Options / Details** |
| --- | --- | --- |
| **A. Characterization of the surveyed dwelling** | | |
| 1 | How many people slept in this dwelling last night? |  |
| 2 | How many of these people belong to the following age groups? | Under 18 years old: |
|  |  | 65 years old or older (Seniors): |
| 3 | Presence of domestic animals (pets)? | [ ] Dogs |
|  |  | [ ] Cats |
|  |  | [ ] Others. Specify: ____________________ |
| 4 | Presence of production animals (livestock)? | [ ] Bovines |
|  |  | [ ] Goats |
|  |  | [ ] Sheep |
|  |  | [ ] Equines |
|  |  | [ ] Others. Specify: ____________________ |
| 5 | Describe how the production animals are kept (one answer for each species/type): | ________________________________________ |
| 6 | Does your dwelling have gaps or holes where you believe bats could enter? | [ ] YES    [ ] NO    [ ] DO NOT KNOW |
| 7 | Does the place where production animals are kept have gaps or holes where you believe bats could enter? | [ ] YES    [ ] NO    [ ] DO NOT KNOW |
| **B. Characterization of the structure associated with bat presence** | | |
| 8 | Have you seen bats inside your dwelling? | [ ] YES    [ ] NO    [ ] DO NOT KNOW |
|  |  | *Location:* ______________________________ |
| 9 | Have you seen areas with bat feces inside your dwelling? | [ ] YES    [ ] NO    [ ] DO NOT KNOW |
|  |  | *Location:* ______________________________ |
| 10 | Have you seen areas with bat feces where your domestic animals (pets) sleep/are kept? | [ ] YES    [ ] NO    [ ] DO NOT KNOW |
|  |  | *Location:* ______________________________ |
| 11 | Have you seen areas with bat feces where your production animals (livestock) are kept? | [ ] YES    [ ] NO    [ ] DO NOT KNOW |
|  |  | *Location:* ______________________________ |
| 12 | Have you seen bats inside the area where your production animals are kept? | [ ] YES    [ ] NO    [ ] DO NOT KNOW |
|  |  | *Location:* ______________________________ |
| 13 | Have you observed contact between bats and your production animals? | [ ] YES    [ ] NO    [ ] DO NOT KNOW |
|  |  | *Location:* ______________________________ |
| 14 | Have you observed contact between bats and your domestic animals (pets)? | [ ] YES    [ ] NO    [ ] DO NOT KNOW |
|  |  | *Location:* ______________________________ |
| 15 | Please characterize the structure housing the bat colony: | *Type:* [ ] Main dwelling [ ] Storage shed/Barn [ ] Other: ______________ |
|  |  | *Distance from main house:* [ ] meters |
|  |  | *Use:* [ ] Single-family [ ] Community |
|  |  | *Size:* [ ] m²    *Age:* [ ] years |
|  |  | *Frequently inhabited?* [ ] YES [ ] NO |
|  |  | *Materials:* [ ] Wood [ ] Concrete [ ] Adobe [ ] Brick [ ] Fiber cement [ ] Zinc/Corrugated metal [ ] Light materials [ ] Other: _____________________ |
| 16 | Since when have you been aware of the presence of bats (years/months)? | ________________________________________ |
| 17 | Have you taken control measures to remove them? | [ ] YES    [ ] NO    [ ] DO NOT KNOW |
|  |  | *Which ones?* __________________________ |
| 18 | If bats are located in other area(s) within your property, please mention them: | ________________________________________ |
| 19 | Do you or any family member frequently visit that/those location(s)? | [ ] YES    [ ] NO    [ ] DO NOT KNOW |
|  |  | *How often?* [ ] 1x/day [ ] 2x/day [ ] 3+x/day [ ] 1x/month |
| 20 | Have you seen bats in places near your property? | [ ] YES    [ ] NO |
|  |  | *Where?* ______________________________ |
| **C. Bat-associated diseases** | | |
| 21 | Did you know that there are diseases that can be transmitted from bats to people? | [ ] YES    [ ] NO    [ ] DO NOT KNOW |
| 22 | If your answer is YES, could you name any? | _______________________________________ |
| 23 | Did you know that there are diseases that can be transmitted from bats to domestic animals (pets)? | [ ] YES    [ ] NO    [ ] DO NOT KNOW |
| 24 | Did you know that there are diseases that can be transmitted from bats to production animals (livestock)? | [ ] YES    [ ] NO    [ ] DO NOT KNOW |
| 25 | Have you made any report to the health authority regarding the presence of bats on your property? | [ ] YES    [ ] NO    [ ] DO NOT KNOW |
| 26 | Are you and/or members of your family vaccinated against rabies? | [ ] YES    [ ] NO    [ ] DO NOT KNOW |
| 27 | In the presence of a bat inside your dwelling, do you know what actions you should take? | [ ] YES    [ ] NO    [ ] DO NOT KNOW |
|  |  | *Briefly explain:* ________________________ |
