# Supplementary material for: Perceptions and public health risks of the bat-human interface in households from fragmented rural landscapes in southern Chile
Source: PLoS One. 2026 Jul 6;21(7):e0353070. doi: 10.1371/journal.pone.0353070 (PMC13336185; doi:10.1371/journal.pone.0353070)
Supplement: S2 Table — (DOCX) [file pone.0353070.s002.docx]

**S2 Table. Coding matrix for the ordinal “human-bat contact level” variable based on questionnaire responses.**

| **Human-Bat Contact Level** | **Epidemiological Risk Gradient** | **Specific Questionnaire Responses / Scenarios Required for Classification** |
| --- | --- | --- |
| **Low** | External presence or indirect environmental evidence only (lowest risk). | Affirmative response to: "If bats are located in other area(s) within your property, please mention them" (when referring to exterior walls, unoccupied barns, or flying nearby).  Must have answered "No" to any indoor presence or physical contact questions. |
| **Medium** | Internal presence within the dwelling implying potential direct exposure (moderate risk). | "Yes" to: "Have you seen bats inside your dwelling?" OR "Have you seen areas with bat feces inside your dwelling?"  Must have answered "No" to observed contact with domestic or production animals, and no reports of physical handling. |
| **High** | Reported direct physical interaction, contact with domestic animals, or unavoidable proximity (highest risk). | "Yes" to: "Have you observed contact between bats and your domestic animals (pets)?" OR "Have you observed contact between bats and your production animals?" OR Qualitative open-ended reports derived from: "Have you taken control measures to remove them?" (e.g., direct handling, capturing, or killing the bat) OR "Please characterize the structure housing the bat colony" (e.g., roosts located directly inside high-traffic living areas like bedrooms or kitchens, defining high frequency access). |
