# Supplementary material for: Perceptions and public health risks of the bat-human interface in households from fragmented rural landscapes in southern Chile
Source: PLoS One. 2026 Jul 6;21(7):e0353070. doi: 10.1371/journal.pone.0353070 (PMC13336185; doi:10.1371/journal.pone.0353070)
Supplement: S1 File — Contingency tables for bivariate associations between household characteristics and human-bat contact levels. (DOCX) [file pone.0353070.s003.docx]

**Contingency tables for bivariate associations between household characteristics and human-bat contact levels.**

This document provides the full data distribution and statistical metrics for the exploratory associations discussed in the main manuscript.

**S3 Table. Association between dog presence and human-bat contact level.**

| **Household characteristic** | **Low Contact (n)** | **Medium Contact (n)** | **High Contact (n)** | **Total (N)** |
| --- | --- | --- | --- | --- |
| **No dog** | 10 (71.4%) | 3 (21.4%) | 1 (7.1%) | 14 |
| **Dog present** | 1 (5.9%) | 8 (47.1%) | 8 (47.1%) | 17 |
| **Total** | 11 | 11 | 9 | 31 |

**Statistical metrics:**

- Pearson's Chi-squared (Monte Carlo p-value): 0.0014
- Cramer's V: 0.694 (95% CI: 0.307–1.000)

**S4 Table. Association between cat presence and human-bat contact level.**

| **Household characteristic** | **Low Contact (n)** | **Medium Contact (n)** | **High Contact (n)** | **Total (N)** |
| --- | --- | --- | --- | --- |
| **No cat** | 10 (55.6%) | 5 (27.8%) | 3 (16.7%) | 18 |
| **Cat present** | 1 (7.7%) | 6 (46.2%) | 6 (46.2%) | 13 |
| **Total** | 11 | 11 | 9 | 31 |

**Statistical metrics:**

- Pearson's Chi-squared (Monte Carlo p-value): 0.0210
- Cramer's V: 0.503 (95% CI: 0.065–0.830)

**S5 Table. Association between vulnerable population (minors/seniors) and human-bat contact level.**

| **Household characteristic** | **Low Contact (n)** | **Medium Contact (n)** | **High Contact (n)** | **Total (N)** |
| --- | --- | --- | --- | --- |
| **Non-vulnerable** | 11 (64.7%) | 3 (17.6%) | 3 (17.6%) | 17 |
| **Vulnerable population** | 0 (0.0%) | 8 (57.1%) | 6 (42.9%) | 14 |
| **Total** | 11 | 11 | 9 | 31 |

**Statistical metrics:**

- Pearson's Chi-squared (Monte Carlo p-value): 0.0014
- Cramer's V: 0.675 (95% CI: 0.286–1.000)

**S6 Table. Association between structural vulnerability (Dwelling) and human-bat contact level.**

| **Household characteristic** | **Low Contact (n)** | **Medium Contact (n)** | **High Contact (n)** | **Total (N)** |
| --- | --- | --- | --- | --- |
| **Not vulnerable** | 1 (25.0%) | 3 (75.0%) | 0 (0.0%) | 4 |
| **Vulnerable dwelling** | 9 (34.6%) | 8 (30.8%) | 9 (34.6%) | 26 |
| **Total** | 10 | 11 | 9 | 30 |

**Statistical metrics:**

- Pearson's Chi-squared (Monte Carlo p-value): 0.2947
- Cramer's V: 0.333 (95% CI: 0.000–0.655)

**S7 Table. Association between bovine presence and human-bat contact level.**

| **Household characteristic** | **Low Contact (n)** | **Medium Contact (n)** | **High Contact (n)** | **Total (N)** |
| --- | --- | --- | --- | --- |
| **No bovines** | 10 (43.5%) | 7 (30.4%) | 6 (26.1%) | 23 |
| **Bovines present** | 1 (12.5%) | 4 (50.0%) | 3 (37.5%) | 8 |
| **Total** | 11 | 11 | 9 | 31 |

**Statistical metrics:**

- Pearson's Chi-squared (Monte Carlo p-value): 0.3413
- Cramer's V: 0.285 (95% CI: 0.000–0.597)
