# Supplementary material for: Perceptions and public health risks of the bat-human interface in households from fragmented rural landscapes in southern Chile
Source: PLoS One. 2026 Jul 6;21(7):e0353070. doi: 10.1371/journal.pone.0353070 (PMC13336185; doi:10.1371/journal.pone.0353070)
Supplement: Attachment — Submitted filename: Response to Editorial Requirements_CV.docx [file pone.0353070.s005.docx]

**Response to Editorial Requirements**

**Title:** Perceptions and public health risks of the bat-human interface in households from fragmented rural landscapes in southern Chile

Dear Editor and Staff,

Thank you for the opportunity to revise our manuscript. We have carefully reviewed the PLOS ONE style requirements and have implemented the following changes to ensure full compliance with the journal's formatting guidelines:

**1. Compliance with Style Requirements and File Naming**

All headings, affiliations, citations and references have been adjusted to meet the journal's guidelines.

**Figure 1 Copyright Compliance:** We clarify that Figure 1 is an original map generated by the authors using R software. The administrative boundary data used as base layers (Comunas.geojson and Regional.geojson) were obtained from official Chilean government repositories, which are in the public domain. No proprietary data or software (e.g., Google Maps, Google Earth) were used in the creation of this figure.

**Figure 2 Copyright Compliance:** We clarify that Figure 2 consists of original photographs taken by the authors during the fieldwork activities for this study. The authors hold the copyright for these images and authorize their publication under the CC BY 4.0 license.

**Figure 3 Copyright Compliance:** We clarify that Figure 3 is an original illustration created by the authors. The silhouettes of the cat, elderly woman, infant, cow, and chicken are in the public domain (CC0 1.0). The dog silhouette (*Canis familiaris dingo*) is an icon by Sam Fraser-Smith (photo) and T. Michael Keesey (silhouette), which is available under a CC BY license. We have updated the figure legend to include the appropriate attribution as required by the license and PLOS ONE policies.

**2. Data Availability Statement**

We have updated our Data Availability Statement in the online submission form and the manuscript. Following the journal's policy, we have removed the co-author as a contact point and replaced it with a non-author institutional contact.

**Revised Data Statement:** "The data supporting the findings of this study are available upon reasonable request for researchers who meet the criteria for access to confidential data. Due to ethical and privacy restrictions regarding human participants in rural communities, data are not publicly available in a repository. Requests for data access can be directed to the Comité Institucional de Cuidado y Uso de Animales (CICUA-UACh) at the following institutional address:

- Contact Name/Body: CICUA-UACh.
- Email: [comiteusoanimales@uach.cl](mailto:comiteusoanimales@uach.cl)

**3. Funding Statement and Competing Interests**

We thank the editor for bringing this to our attention. However, we would like to clarify that author Annia Rodríguez-San Pedro’s (AR-SP) affiliation was inadvertently listed as commercial in the previous version. Her participation in this study corresponds strictly to her academic affiliation. We have updated her affiliation in the revised manuscript and the online submission form to accurately reflect this: Centro de Investigación e Innovación para el Cambio Climático, Facultad de Ciencias, Universidad Santo Tomás, Santiago, Chile.

Consequently, Bioecos EIRL did not provide salary or any other financial support for this study, and there are no commercial competing interests to declare. We provide our updated statements below:

Updated Funding Statement: "This study was supported by the National Agency for Research and Development (ANID) through the ANILLO project ATE220062. The funders had no role in study design, data collection and analysis, decision to publish, or preparation of the manuscript."

Updated Competing Interests Statement: "The authors have declared that no competing interests exist”.

**Reviewer 1:**

1. The survey included a question asking people if they're vaccinated against rabies. I would have liked to see those responses summarized in the paper. Going a step further: it would be valuable to also show information on population rabies vaccination rates in the Los Ríos and Los Lagos regions. This could allow you to draw an interesting comparison of how the sample vaccination rates compare to the population vaccination rates.

**Author´s response:** We thank the reviewer for this valuable suggestion. We have now included the summarized responses regarding human rabies vaccination in the results section of the manuscript. Out of 69 surveyed households, only 13.0% (n=9) reported that they or a family member had been vaccinated against rabies, while 79.7% (n=55) reported not being vaccinated (the remaining 7.2% did not know or left the question blank).

Regarding the suggestion to compare our sample rates with population rabies vaccination rates in the Los Ríos and Los Lagos regions, it is important to clarify a key aspect of public health policy in Chile. Human rabies vaccination is not part of the universal routine immunization schedule. According to the Chilean Ministry of Health (MINSAL), human rabies vaccines are strictly administered as Post-Exposure Prophylaxis (PEP) for individuals bitten by suspect animals (such as dogs or wild mammals) or exposed to bats, and as pre-exposure prophylaxis for specific high-risk occupational groups (Ministerio de Salud, 2014).

Consequently, a "population vaccination rate" or coverage metric does not exist for humans in Chile as it does for routine vaccines. The 13% of respondents in our survey who reported being vaccinated likely represent individuals who received PEP at some point in their lives due to an animal bite or bat exposure. However, to provide the regional context the reviewer rightly points out, we have added official data to the discussion section regarding the incidence of PEP administration. In 2023, 2,453 first doses of the antirabies vaccine were administered in the Los Ríos region, and 4,702 first doses were administered in the Los Lagos region (Luchsinger et al.,2025). We believe this contextualizes the actual dynamics of rabies vaccination in these territories.

2. You mention that people have a low perception of the actual risk of rabies transmission to humans in the country. But, I was left wondering, what is the actual risk of rabies transmission? Again, could you provide statistics for rabies rates in the populations of the two regions considered in the paper? This would help a reader conceptualize how big of a risk rabies actually is.

**Author´s response:** We appreciate the reviewer’s request for clarification, as it highlights a crucial epidemiological paradox in our study area. To address this, it is important to state that the actual incidence rate of human rabies in Chile and specifically in the Los Ríos and Los Lagos regions is practically zero.

Chile was declared free of human rabies transmitted by dogs decades ago, with the last canine-variant case reported in 1972 (Ministerio de Salud, 2021; Luchsinger et al., 2025). Since the discovery of bats as the main reservoir in 1985, only two human rabies cases have been reported nationally: one in 1996 (from a bat variant) and another in 2013 (an encephalitis case without virological confirmation) (Luchsinger et al., 2025; Alegría-Moran et al., 2017). Neither of these cases occurred in the Los Ríos or Los Lagos regions.

Therefore, providing a statistical 'rabies rate' for the human populations in our studied regions would yield 0 cases per 100,000 inhabitants. This extremely low disease incidence is precisely the root of the problem: because people do not see or hear about human rabies cases, they do not perceive bats as a tangible threat. However, this zero human incidence occurs despite the virus actively circulating in the environment. Longitudinal data from the National Rabies Surveillance Program (Instituto de Salud Pública - ISP) shows that rabies positivity among tested bats nationwide consistently ranges between 7% and 10%, reaching 8.7% in 2023 and 8.6% in 2024. Furthermore, the virus is highly active in our specific study area; for instance, bats tested in the Los Lagos region recorded a 17.4% positivity rate in 2023 (ISP, 2023; 2024).

As our study shows, the exposure hazard is high due to frequent human-bat cohabitation, and this risk is actively managed by the health system through the high regional demand for Post-Exposure Prophylaxis (PEP) that we discussed in the previous comment. We have added a paragraph to the discussion to clarify the actual incidence of the disease, the sustained viral prevalence in local bat populations, and how this near-absence of human cases drives the low risk perception.

3. You mentioned that dwellings/structures in the immediate vicinity were visited. I'm wondering, was there any specific threshold distance beyond which you did not consider dwellings/structures as eligible? Also, was there a target or maximum number of dwellings/structures you targeted per each sampling point?

**Author´s response:** We thank the reviewer for this question, which allows us to clarify our sampling approach at the household level. We did not establish a specific threshold distance in meters, nor a maximum number of targeted structures per sampling point. Instead, our operational boundary was the fenced perimeter of each selected property.

As it is common in rural areas of southern Chile for a single property to contain more than one building (e.g., a main house, a secondary house for extended family, sheds, or even small community structures like a local church where the homeowner is the pastor), all structures within the property limits were considered eligible and visited. We have updated the Methods section in the revised manuscript to clearly reflect this operational definition.

4. You have a sentence about excluding surveys if fewer than 75% of questions were answered or if critical missing information could not be resolved after three attempted contacts. Please provide readers with the specific number of surveys that fell into those categories and thus were omitted.

**Author´s response:** We thank the reviewer for pointing out this omission. We agree that providing the exact number of excluded surveys provides greater transparency to our data collection process. As requested, we have updated the manuscript to explicitly state that a total of 72 surveys were initially obtained, of which 3 did not meet the data quality criteria and were therefore omitted, leaving 69 correctly completed surveys for the final analysis.

5. You settled on using three contact levels (low, medium, high). That makes sense, but it would be great if you provided readers a little more information on how exactly you settled on using these three levels, as well as what sorts of specific rates constitute things like "high frequency access" to bats.

**Author´s response:** We thank the reviewer for this constructive comment. We agree that the rationale behind our contact level categorization needed more clarity for the readers. The three levels (low, medium, high) were established to reflect a gradient of epidemiological risk, moving from mere environmental overlap (lowest risk) to potential indoor exposure (medium risk), and finally to direct physical exposure or unavoidable proximity (highest risk, direct contact).

Regarding "high frequency access", since our cross-sectional survey did not measure a specific numerical rate (e.g., number of interactions per week), we realize the phrasing could be misinterpreted. We used this term to describe qualitative scenarios where the spatial overlap makes recurrent encounters practically unavoidable, for instance, when a bat colony is roosting directly inside high-traffic living areas (such as bedrooms or kitchens), or when residents or pets repeatedly interact with bats while trying to remove them from the living space. We have expanded the methodology section to explicitly detail what constitutes each level with concrete examples, and we have clarified the definition of “high frequency access”.

6. The action category variable includes three groups: negative/aggressive, neutral/evasive, and precautionary/informative. However, I'm concerned that the breakdown may not be clean. For example, what if someone is the type of person that engages in preventative measures (thus falling into the precautionary/informative group), but then a bat makes its way into their house anyway, and then they react in a negative/aggressive way (thus also falling into the negative/aggressive category)? This would mean that the categories aren't mutually exclusive.

**Author´s response:** We thank the reviewer for this perceptive comment. We realize that our use of the phrase 'preventive measures' when defining the “precautionary/informative” category caused unintended confusion. To clarify, this variable was not designed to classify a respondent's general lifestyle or prior structural prevention habits (e.g., sealing gaps in the house). Instead, it exclusively measures the respondent's primary, immediate reaction during the specific event of finding a bat indoors.

Because the open-ended responses focused solely on the immediate action taken during the encounter, the categories are mutually exclusive. Each of the 18 respondents described a primary course of action for that specific moment, allowing them to be classified into an unique group. To prevent the exact confusion the reviewer correctly pointed out, we have rephrased the definition of the “precautionary/informative” category in the methodology, replacing “preventive measures” with “safe containment or precautionary actions during the encounter”. We believe this clearly separates the immediate reaction to an indoor bat from general preventative household maintenance.

7. In Figure 1, given that you use color, I would recommend making water appear a light blue in the map.

**Author´s response:** We thank the reviewer for this recommendation. We agree that this change improves the visual clarity of the map. Figure 1 has been updated accordingly, and the water is now displayed in light blue.

8. You provide summary statistics for people with dogs and people with cats. I'm wondering whether it might be interesting to distinguish this a little more specifically: people with dogs only, people with cats only, and people with both dogs and cats.

**Author´s response:** We thank the reviewer for this suggestion. We agree that breaking down pet ownership into mutually exclusive categories provides a much clearer and interesting picture of the domestic animal interface in our study area.

Based on our data, out of the 69 surveyed households, 44.9% (n=31) owned both dogs and cats, 20.3% (n=14) owned only dogs, and 5.8% (n=4) owned only cats, accounting for the 49 households that reported having pets. We have updated the 'Household sample and baseline characteristics' subsection in the Results to include this detailed and specific breakdown, replacing the previous summary statistics.

**Reviewer 2:**

1. Clarification of the derived contact-level variable

The ordinal “human-bat contact level” variable is central to the analysis and interpretation of the results. However, the manuscript should describe more explicitly how this variable was constructed from the questionnaire responses. I suggest adding a supplementary coding table indicating which specific answers were used to classify households as low, medium, or high contact. This would improve reproducibility and allow readers to better understand the analytical approach.

**Author´s response:** We thank the reviewer for this constructive suggestion, which indeed enhances the transparency and reproducibility of our study. In response to a related comment raised by Reviewer 1 regarding the definition of these categories, we have already expanded the text in the Methods section to explicitly detail the epidemiological risk gradient defining the “low”, “medium”, and “high” contact levels, and clarified qualitative terms such as “high frequency access”.

Furthermore, we completely agree with your suggestion to provide a coding matrix. We have now created and referenced a new supplementary table (S2 Table) that explicitly maps the specific questionnaire responses and reported scenarios used to classify each household into its respective contact level. We believe this addition provides the exact clarity requested.

2. Clarification of the knowledge score

The health-risk knowledge score is useful as a simple descriptive indicator, but it should be presented as such. The score appears to capture whether respondents recognize potential disease transmission from bats to humans, pets, and livestock, but it does not necessarily measure depth, accuracy, or practical applicability of knowledge. The authors should clarify this limitation and avoid implying that the score is a validated measure of health-risk knowledge.

**Author´s response:** We thank the reviewer for this perceptive observation. We completely agree that the health-risk knowledge score should be interpreted strictly as a basic descriptive indicator rather than a validated psychometric tool. Our intention was solely to capture the breadth of awareness across the three epidemiological interfaces (humans, pets, and livestock), but we acknowledge that it does not reflect the depth, accuracy, or practical applicability of the respondents' knowledge.

To prevent any misinterpretation and to clearly state this limitation, we have added a clarifying sentence in the Methodology section when the score is first introduced, and we have explicitly addressed this constraint in the limitations paragraph of the Discussion section

3. Interpretation of public health risk

The manuscript refers to “public health risks”, which is appropriate as a broad framing. However, the study does not directly measure pathogen presence, confirmed bite or scratch events, clinical disease, or quantitative infection risk. Therefore, some statements should be slightly moderated to emphasize that the study provides evidence of exposure opportunities, risk-related household conditions, and perceived risk, rather than direct measurement of pathogen transmission or disease risk.

**Author´s response:** We thank the reviewer for this important and precise observation. We completely agree that while the broad framing of “public health risks” is relevant, our study design was not intended to directly measure pathogen presence, confirm specific clinical disease events, or quantify actual infection rates.

Following your recommendation, we have carefully reviewed the manuscript and moderated our statements throughout the text (e.g., changing “disease risk” to “exposure opportunities” or “potential transmission pathways” where appropriate). Furthermore, to ensure readers correctly interpret our results, we have explicitly stated this distinction in the limitations section of the Discussion. We emphasize that our findings provide evidence of exposure opportunities, risk-related structural vulnerabilities, and perceived risk, which serve as the foundational prerequisites for potential spillover, rather than direct measurements of pathogen transmission.

4. Statistical analysis

The statistical approach is appropriate for the descriptive and exploratory nature of the study. The use of descriptive statistics, confidence intervals for proportions, Fisher’s exact test or chi-square tests with Monte Carlo simulation for small cell counts, and Spearman’s correlation is reasonable. However, the authors should consider presenting complete contingency tables and, where possible, effect-size estimates with confidence intervals, rather than relying mainly on p-values. The exploratory nature of the associations should also be clearly stated.

**Author´s response:** We thank the reviewer for this constructive feedback, which undoubtedly strengthens the statistical rigor and transparency of our manuscript. We agree that relying solely on p-values is insufficient and that providing effect sizes and full contingency tables offers a more comprehensive understanding of our data.

In the revised manuscript, we have made the following modifications: 1. We have explicitly stated the exploratory nature of these bivariate associations in the 'Statistical Analyses' subsection, clarifying that they are intended for hypothesis generation rather than causal inference. 2. We have calculated effect-size estimates (Cramer's V for categorical associations) along with their 95% confidence intervals. 3. We have created a new Supplementary Table (S3 Table) that presents the complete contingency tables for all evaluated associations (e.g., contact level vs. presence of pets, vulnerable residents, and structural vulnerability) alongside their respective effect sizes and p-values. The Results section has been updated to reference this new table.

5. Data availability

The current Data Availability Statement indicates that the dataset is not publicly available due to privacy restrictions related to the exact location of rural dwellings and that data are available from the corresponding author upon reasonable request. This does not fully meet the expectation that the data underlying the findings be made available without restriction, except under clearly justified ethical or legal constraints. I recommend that the authors deposit a de-identified dataset in a public repository, removing exact coordinates or replacing them with aggregated or anonymized spatial variables. If public sharing is not possible, the restrictions should be described in more detail, including the institutional procedure for requesting access.

**Author´s response:** We thank the reviewer for this recommendation regarding data transparency. A very similar concern was raised by the Academic Editor during the initial evaluation. Because our study involves human participants in small, geographically specific rural communities, stripping exact coordinates does not completely guarantee anonymity, as the combination of specific household demographics, pet ownership, and structural variables could still lead to deductive disclosure. Therefore, under the guidelines of our ethics committee, the dataset cannot be deposited in a fully public repository.

However, to fully comply with the journal's policies for restricted data, and following the Editor's instructions, we have updated the Data Availability Statement in both the online submission form and the manuscript. We have described the restrictions in more detail and replaced the author contact with a formal, non-author institutional contact (the Scientific Ethics Committee) to handle reasonable requests for data access.

6. Ethics statement

The study involved surveys with human participants. The ethics statement indicates approval by an Institutional Committee for the Care and Use of Animals in Research. The authors should clarify whether this committee had formal authority to review human-subject research involving surveys. If applicable, approval or exemption from an appropriate human research ethics committee should be reported.

**Author´s response:** We thank the reviewer for raising this valid point and allowing us to clarify the ethical approval process. This study was conducted as part of a larger overarching ecological project (ANILLO ATE220062) whose primary objective involves the capture and sampling of wild bats and rodents. Consequently, the primary ethical evaluation was conducted by the Institutional Committee for the Care and Use of Animals in Research (CICUA) of the Universidad Austral de Chile (protocol code 510/2023). However, the household survey and the informed consent procedures were submitted as a formal amendment to this overarching animal protocol. The CICUA formally reviewed and approved the application of this questionnaire and the human informed consent forms on January 10, 2024, evaluating it as a supplementary, low-risk data collection tool applied in the exact same households where the animal sampling occurred. We have clarified this context in the Ethics Statement of the revised manuscript to address your concern.

7.Language and terminology

The manuscript is intelligible and written in generally understandable English. Nevertheless, it would benefit from some English language editing. Some expressions should be corrected or refined, for example “Emerging of infectious diseases” (should say emergence), “Whitin”, and “natural refugees”. The term “sanitary risk” should also be reconsidered in several places; depending on the context, “health risk”, “public health risk”, or “risk of exposure” may be more appropriate. Please also standardize terminology such as “Los Ríos”, “southern Chile”, “households”, “dwellings”, and “bat presence” throughout the manuscript.

**Author´s response:** We thank the reviewer for the careful reading of our manuscript and for highlighting these typographical and terminology issues. We have thoroughly reviewed and edited the English language throughout the revised manuscript. Specifically, we have corrected “Emerging of infectious diseases” to “The emergence of infectious diseases”, “Whitin” to “Within”, and “natural refugees” to “natural refuges”. Furthermore, we have carefully reconsidered the use of the term “sanitary risk” and replaced it with “public health risk”, “health risk”, or “risk of exposure”, depending on the specific context of the sentence. Finally, we have standardized the terminology for “Los Ríos”, “southern Chile”, “households”, “dwellings”, and “bat presence” across the entire document to ensure consistency.

We believe these adjustments address all the formatting requirements mentioned in the editorial letter. We remain at your disposal for any further technical or scientific clarifications.

Sincerely,

**Cristobal Verdugo, PhD** Corresponding Author

Universidad Austral de Chile
