# Supplementary material for: Perceptions and public health risks of the bat-human interface in households from fragmented rural landscapes in southern Chile
Source: PLoS One. 2026 Jul 6;21(7):e0353070. doi: 10.1371/journal.pone.0353070 (PMC13336185; doi:10.1371/journal.pone.0353070)
Supplement: Peer review history

# Peer review: Decision Letter 0

**DOI:** 10.1371/journal.pone.0353070.r001

**Authors:** Daniel Oluwayelu

12 May 2026

PONE-D-26-17161

Perceptions and public health risks of the bat-human interface in households from fragmented rural landscapes in Southern Chile.

PLOS One

Dear Dr.  Hernandez,

Thank you for submitting your manuscript to PLOS ONE. After careful consideration, we feel that it has merit but does not fully meet PLOS ONE’s publication criteria as it currently stands. Therefore, we invite you to submit a revised version of the manuscript that addresses the points raised during the review process.

Please submit your revised manuscript by Jun 26 2026 11:59PM. If you will need more time than this to complete your revisions, please reply to this message or contact the journal office at plosone@plos.org. When you're ready to submit your revision, log on to https://www.editorialmanager.com/pone/ and select the 'Submissions Needing Revision' folder to locate your manuscript file.

Please include the following items when submitting your revised manuscript:

- A letter that responds to each point raised by the academic editor and reviewer(s). You should upload this letter as a separate file labeled 'Response to Reviewers'.
- A marked-up copy of your manuscript that highlights changes made to the original version. You should upload this as a separate file labeled 'Revised Manuscript with Track Changes'.
- An unmarked version of your revised paper without tracked changes. You should upload this as a separate file labeled 'Manuscript'.

If you would like to make changes to your financial disclosure, please include your updated statement in your cover letter. Guidelines for resubmitting your figure files are available below the reviewer comments at the end of this letter.

If applicable, we recommend that you deposit your laboratory protocols in protocols.io to enhance the reproducibility of your results. Protocols.io assigns your protocol its own identifier (DOI) so that it can be cited independently in the future. For instructions see: https://journals.plos.org/plosone/s/submission-guidelines#loc-laboratory-protocols. Additionally, PLOS ONE offers an option for publishing peer-reviewed Lab Protocol articles, which describe protocols hosted on protocols.io. Read more information on sharing protocols at https://plos.org/protocols?utm_medium=editorial-email&utm_source=authorletters&utm_campaign=protocols.

As the corresponding author, your ORCID iD is verified in the submission system and will appear in the published article. PLOS supports the use of ORCID, and we encourage all coauthors to register for an ORCID iD and use it as well. Please encourage your coauthors to verify their ORCID iD within the submission system before final acceptance, as unverified ORCID iDs will not appear in the published article. *Only*  the individual author can complete the verification step; PLOS staff *cannot*  verify ORCID iDs on behalf of authors.

We look forward to receiving your revised manuscript.

Kind regards,

Daniel Oladimeji Oluwayelu, D.V.M., M.Sc., Ph.D.

Academic Editor

PLOS One

Journal Requirements:

When submitting your revision, we need you to address these additional requirements.

1.Please ensure that your manuscript meets PLOS ONE's style requirements, including those for file naming. The PLOS ONE style templates can be found at

https://journals.plos.org/plosone/s/file?id=wjVg/PLOSOne_formatting_sample_main_body.pdf and

https://journals.plos.org/plosone/s/file?id=ba62/PLOSOne_formatting_sample_title_authors_affiliations.pdf

2. In the online submission form you indicate that your data is not available for proprietary reasons and have provided a contact point for accessing this data. Please note that your current contact point is a co-author on this manuscript. According to our Data Policy, the contact point must not be an author on the manuscript and must be an institutional contact, ideally not an individual. Please revise your data statement to a non-author institutional point of contact, such as a data access or ethics committee, and send this to us via return email. Please also include contact information for the third party organization, and please include the full citation of where the data can be found.

3. Thank you for stating the following in the Competing Interests section:

“The authors declare that they have no competing interests.”

We note that one or more of the authors are employed by a commercial company: Bioecos EIRL, Apoquindo 6410, Oficina 1004, Las Condes, Región Metropolitana, Chile

1.        Please provide an amended Funding Statement declaring this commercial affiliation, as well as a statement regarding the Role of Funders in your study. If the funding organization did not play a role in the study design, data collection and analysis, decision to publish, or preparation of the manuscript and only provided financial support in the form of authors' salaries and/or research materials, please review your statements relating to the author contributions, and ensure you have specifically and accurately indicated the role(s) that these authors had in your study. You can update author roles in the Author Contributions section of the online submission form.

Please also include the following statement within your amended Funding Statement.

“The funder provided support in the form of salaries for authors [insert relevant initials], but did not have any additional role in the study design, data collection and analysis, decision to publish, or preparation of the manuscript. The specific roles of these authors are articulated in the ‘author contributions’ section.”

If your commercial affiliation did play a role in your study, please state and explain this role within your updated Funding Statement.

2. Please also provide an updated Competing Interests Statement declaring this commercial affiliation along with any other relevant declarations relating to employment, consultancy, patents, products in development, or marketed products, etc.

Within your Competing Interests Statement, please confirm that this commercial affiliation does not alter your adherence to all PLOS ONE policies on sharing data and materials by including the following statement: "This does not alter our adherence to  PLOS ONE policies on sharing data and materials.” (as detailed online in our guide for authors http://journals.plos.org/plosone/s/competing-interests) . If this adherence statement is not accurate and  there are restrictions on sharing of data and/or materials, please state these. Please note that we cannot proceed with consideration of your article until this information has been declared.

Please include both an updated Funding Statement and Competing Interests Statement in your cover letter. We will change the online submission form on your behalf.

4. We note that Figure 1 in your submission contain map images which may be copyrighted. All PLOS content is published under the Creative Commons Attribution License (CC BY 4.0), which means that the manuscript, images, and Supporting Information files will be freely available online, and any third party is permitted to access, download, copy, distribute, and use these materials in any way, even commercially, with proper attribution. For these reasons, we cannot publish previously copyrighted maps or satellite images created using proprietary data, such as Google software (Google Maps, Street View, and Earth). For more information, see our copyright guidelines: http://journals.plos.org/plosone/s/licenses-and-copyright.

We require you to either (1) present written permission from the copyright holder to publish these figures specifically under the CC BY 4.0 license, or (2) remove the figures from your submission:

1. You may seek permission from the original copyright holder of Figure 1 to publish the content specifically under the CC BY 4.0 license.

We recommend that you contact the original copyright holder with the Content Permission Form (http://journals.plos.org/plosone/s/file?id=7c09/content-permission-form.pdf) and the following text:

“I request permission for the open-access journal PLOS ONE to publish XXX under the Creative Commons Attribution License (CCAL) CC BY 4.0 (http://creativecommons.org/licenses/by/4.0/). Please be aware that this license allows unrestricted use and distribution, even commercially, by third parties. Please reply and provide explicit written permission to publish XXX under a CC BY license and complete the attached form.”

Please upload the completed Content Permission Form or other proof of granted permissions as an "Other" file with your submission.

In the figure caption of the copyrighted figure, please include the following text: “Reprinted from [ref] under a CC BY license, with permission from [name of publisher], original copyright [original copyright year].”

2. If you are unable to obtain permission from the original copyright holder to publish these figures under the CC BY 4.0 license or if the copyright holder’s requirements are incompatible with the CC BY 4.0 license, please either i) remove the figure or ii) supply a replacement figure that complies with the CC BY 4.0 license. Please check copyright information on all replacement figures and update the figure caption with source information. If applicable, please specify in the figure caption text when a figure is similar but not identical to the original image and is therefore for illustrative purposes only.

The following resources for replacing copyrighted map figures may be helpful:

USGS National Map Viewer (public domain): http://viewer.nationalmap.gov/viewer/

The Gateway to Astronaut Photography of Earth (public domain): http://eol.jsc.nasa.gov/sseop/clickmap/

Maps at the CIA (public domain): https://www.cia.gov/library/publications/the-world-factbook/index.html and https://www.cia.gov/library/publications/cia-maps-publications/index.html

NASA Earth Observatory (public domain): http://earthobservatory.nasa.gov/

Landsat: http://landsat.visibleearth.nasa.gov/

USGS EROS (Earth Resources Observatory and Science (EROS) Center) (public domain): http://eros.usgs.gov/#

Natural Earth (public domain): http://www.naturalearthdata.com/

5. We note that Figure 2 and 3 in your submission contain copyrighted images. All PLOS content is published under the Creative Commons Attribution License (CC BY 4.0), which means that the manuscript, images, and Supporting Information files will be freely available online, and any third party is permitted to access, download, copy, distribute, and use these materials in any way, even commercially, with proper attribution. For more information, see our copyright guidelines: http://journals.plos.org/plosone/s/licenses-and-copyright.

We require you to either (1) present written permission from the copyright holder to publish these figures specifically under the CC BY 4.0 license, or (2) remove the figures from your submission:

1. You may seek permission from the original copyright holder of Figure 2 and 3 to publish the content specifically under the CC BY 4.0 license.

We recommend that you contact the original copyright holder with the Content Permission Form (http://journals.plos.org/plosone/s/file?id=7c09/content-permission-form.pdf) and the following text:

“I request permission for the open-access journal PLOS ONE to publish XXX under the Creative Commons Attribution License (CCAL) CC BY 4.0 (http://creativecommons.org/licenses/by/4.0/). Please be aware that this license allows unrestricted use and distribution, even commercially, by third parties. Please reply and provide explicit written permission to publish XXX under a CC BY license and complete the attached form.”

Please upload the completed Content Permission Form or other proof of granted permissions as an "Other" file with your submission.

In the figure caption of the copyrighted figure, please include the following text: “Reprinted from [ref] under a CC BY license, with permission from [name of publisher], original copyright [original copyright year].”

2. If you are unable to obtain permission from the original copyright holder to publish these figures under the CC BY 4.0 license or if the copyright holder’s requirements are incompatible with the CC BY 4.0 license, please either i) remove the figure or ii) supply a replacement figure that complies with the CC BY 4.0 license. Please check copyright information on all replacement figures and update the figure caption with source information. If applicable, please specify in the figure caption text when a figure is similar but not identical to the original image and is therefore for illustrative purposes only.

6. If the reviewer comments include a recommendation to cite specific previously published works, please review and evaluate these publications to determine whether they are relevant and should be cited. There is no requirement to cite these works unless the editor has indicated otherwise.

7. Please review your reference list to ensure that it is complete and correct. If you have cited papers that have been retracted, please include the rationale for doing so in the manuscript text, or remove these references and replace them with relevant current references. Any changes to the reference list should be mentioned in the rebuttal letter that accompanies your revised manuscript. If you need to cite a retracted article, indicate the article’s retracted status in the References list and also include a citation and full reference for the retraction notice.

[Note: HTML markup is below. Please do not edit.]

Reviewers' comments:

Reviewer's Responses to Questions

**Comments to the Author**

1. Is the manuscript technically sound, and do the data support the conclusions?

The manuscript must describe a technically sound piece of scientific research with data that supports the conclusions. Experiments must have been conducted rigorously, with appropriate controls, replication, and sample sizes. The conclusions must be drawn appropriately based on the data presented.

Reviewer #1: Yes

Reviewer #2: Yes

**********

2. Has the statistical analysis been performed appropriately and rigorously?

Reviewer #1: Yes

Reviewer #2: Yes

**********

3. Have the authors made all data underlying the findings in their manuscript fully available?

The PLOS Data policy requires authors to make all data underlying the findings described in their manuscript fully available without restriction, with rare exception (please refer to the Data Availability Statement in the manuscript PDF file). The data should be provided as part of the manuscript or its supporting information, or deposited to a public repository. For example, in addition to summary statistics, the data points behind means, medians and variance measures should be available. If there are restrictions on publicly sharing data—e.g. participant privacy or use of data from a third party—those must be specified.

Reviewer #1: No

Reviewer #2: No

**********

4. Is the manuscript presented in an intelligible fashion and written in standard English?

PLOS ONE does not copyedit accepted manuscripts, so the language in submitted articles must be clear, correct, and unambiguous. Any typographical or grammatical errors should be corrected at revision, so please note any specific errors here.

Reviewer #1: Yes

Reviewer #2: Yes

**********

5. Review Comments to the Author

Please use the space provided to explain your answers to the questions above. You may also include additional comments for the author, including concerns about dual publication, research ethics, or publication ethics. (Please upload your review as an attachment if it exceeds 20,000 characters)

Reviewer #1: In this manuscript, the authors examine rural residents' perceptions of bats, including perceived risks, across the three interfaces of humans, pets, and livestock. I enjoyed reading the manuscript, which in my opinion is well done. Below, I split my comments into major comments and minor comments.

MAJOR COMMENTS

1. The survey included a question asking people if they're vaccinated against rabies. I would have liked to see those responses summarized in the paper. Going a step further: it would be valuable to also show information on population rabies vaccination rates in the Los Ríos and Los Lagos regions. This could allow you to draw an interesting comparison of how the sample vaccination rates compare to the population vaccination rates.

2. You mention that people have a low perception of the actual risk of rabies transmission to humans in the country. But, I was left wondering, what is the actual risk of rabies transmission? Again, could you provide statistics for rabies rates in the populations of the two regions considered in the paper? This would help a reader conceptualize how big of a risk rabies actually is.

3. You mentioned that dwellings/structures in the immediate vicinity were visited. I'm wondering, was there any specific threshold distance beyond which you did not consider dwellings/structures as eligible? Also, was there a target or maximum number of dwellings/structures you targeted per each sampling point?

4. You have a sentence about excluding surveys if fewer than 75% of questions were answered or if critical missing information could not be resolved after three attempted contacts. Please provide readers with the specific number of surveys that fell into those categories and thus were omitted.

5. You settled on using three contact levels (low, medium, high). That makes sense, but it would be great if you provided readers a little more information on how exactly you settled on using these three levels, as well as what sorts of specific rates constitute things like "high frequency access" to bats.

6. The action category variable includes three groups: negative/aggressive, neutral/evasive, and precautionary/informative. However, I'm concerned that the breakdown may not be clean. For example, what if someone is the type of person that engages in preventative measures (thus falling into the precautionary/informative group), but then a bat makes its way into their house anyway, and then they react in a negative/aggressive way (thus also falling into the negative/aggressive category)? This would mean that the categories aren't mutually exclusive.

7. In Figure 1, given that you use color, I would recommend making water appear a light blue in the map.

8. You provide summary statistics for people with dogs and people with cats. I'm wondering whether it might be interesting to distinguish this a little more specifically: people with dogs only, people with cats only, and people with both dogs and cats.

9. In general, I thought the overall writing throughout the manuscript was of pretty high quality. However, there is a drop-off in the Discussion. In my minor comments below, I offer some suggestions for improvements.

MINOR COMMENTS

Line 45: Remove the "of"

Line 53: "Whitin" is misspelled

Line 92: Need a space between "information" and "[28]"

Line 99: Consider making "perception" plural

Line 131: The "a" should be an "and"

Line 135: Recommend clarifying that age refers to age of the dwelling. Also, recommend removing the "and" in this line given that there's an "and" before the last term of the list in line 136.

Line 161: "with three groups" would sound better than "into three groups" (especially because "into" was already used just a bit earlier in the sentence)

Line 168: Recommend this rephrasing: "… and construction older than 20 years."

Table 1: Recommend having the "Presence of Domestic Animals (N=69 households)" header go across the full width of the table rather than being contained just in the first column

Line 222: The comma in this line would be better as a colon

Table 2: Try to get the "Total Evaluated (N)" header to fit on one line

Line 227: You start this line with an incomplete sentence

Line 232: It's said that bat colonies "were found". This is written in passive voice and makes it unclear who was doing the finding. Was it residents? Or interviewers?

Line 258: Remove the "was"

Line 308: Remove the comma

Line 317: Change "bringing into" into "bringing bats into"

Lines 318-319: Need better parallel structure in this sentence. You say "pets can act […] drawing […] are recognized". Instead, consider: "pets can act […], drawing […] and serving as recognized intermediary hosts […]". Also, the "linking wildlife and humans" part might be better as "thereby linking wildlife and humans"

Line 327: No comma

Lines 330-332: Rewrite this sentence for clarity (sentence that starts with "In contrast, …")

Lines 337-341: Rewrite this sentence for clarity (sentence that starts with "Thus, rural…"). It gets convoluted towards the end.

Line 342: Use "disconnect" rather than "disconnection"

Line 343: Need a comma between "[20,21]" and "was observed"

Line 344: Make "bat" plural

Line 346: The "representing a public health gap" makes this sentence a bit overwhelming. Consider rewriting.

Lines 349-352: The subject of the sentence is "residents", and in the first part of the sentence you use "to recognize". However, later in the sentence you switch to "underestimating". Verb tense agreement would be better as "to underestimate". Consider rewriting this sentence along these lines.

Line 355: Instead of "…, dominated…", consider "…, which are instead dominated…"

Line 358: Needs to be "accounting for"

Line 359: You start this sentence with "further", after having started the prior sentence with "however", and the sentence before that with "thus". Words like these are often not necessary and can distract from the material.

Line 366-367: The "lacking consistent…" end part of this sentence becomes overwhelming. Consider splitting this into its own sentence.

Line 368: Remove the second comma

Line 369: Add a comma between "exposure" and "are"

Line 378: The "Making it difficult…" is an incomplete sentence

Line 536: Replace "applied to" with "administered to"

S1 Appendix: In the survey question table, I would recommend top-aligning the question numbers (rather than having them aligned in the middle as currently)

Reviewer #2: This manuscript addresses a relevant and under-described One Health and public health interface in rural southern Chile: the coexistence of synanthropic bats, humans, companion animals, and livestock in fragmented rural landscapes. Overall, the study is technically sound for a descriptive and exploratory cross-sectional design, and the data generally support the main conclusions. The survey instrument is consistent with the objectives of the study and includes relevant information on household characteristics, structural features, presence of pets and livestock, bat presence, guano in human and animal areas, observed bat–animal contact, control measures, reporting to authorities, rabies vaccination, and knowledge of bat-associated diseases.

The manuscript is potentially suitable for publication after minor revision. I provide the following comments to improve clarity, reproducibility, and interpretation.

1. Clarification of the derived contact-level variable

The ordinal “human-bat contact level” variable is central to the analysis and interpretation of the results. However, the manuscript should describe more explicitly how this variable was constructed from the questionnaire responses. I suggest adding a supplementary coding table indicating which specific answers were used to classify households as low, medium, or high contact. This would improve reproducibility and allow readers to better understand the analytical approach.

2. Clarification of the knowledge score

The health-risk knowledge score is useful as a simple descriptive indicator, but it should be presented as such. The score appears to capture whether respondents recognize potential disease transmission from bats to humans, pets, and livestock, but it does not necessarily measure depth, accuracy, or practical applicability of knowledge. The authors should clarify this limitation and avoid implying that the score is a validated measure of health-risk knowledge.

3. Interpretation of public health risk

The manuscript refers to “public health risks”, which is appropriate as a broad framing. However, the study does not directly measure pathogen presence, confirmed bite or scratch events, clinical disease, or quantitative infection risk. Therefore, some statements should be slightly moderated to emphasize that the study provides evidence of exposure opportunities, risk-related household conditions, and perceived risk, rather than direct measurement of pathogen transmission or disease risk.

4. Statistical analysis

The statistical approach is appropriate for the descriptive and exploratory nature of the study. The use of descriptive statistics, confidence intervals for proportions, Fisher’s exact test or chi-square tests with Monte Carlo simulation for small cell counts, and Spearman’s correlation is reasonable. However, the authors should consider presenting complete contingency tables and, where possible, effect-size estimates with confidence intervals, rather than relying mainly on p-values. The exploratory nature of the associations should also be clearly stated.

5. Data availability

The current Data Availability Statement indicates that the dataset is not publicly available due to privacy restrictions related to the exact location of rural dwellings and that data are available from the corresponding author upon reasonable request. This does not fully meet the expectation that the data underlying the findings be made available without restriction, except under clearly justified ethical or legal constraints. I recommend that the authors deposit a de-identified dataset in a public repository, removing exact coordinates or replacing them with aggregated or anonymized spatial variables. If public sharing is not possible, the restrictions should be described in more detail, including the institutional procedure for requesting access.

6. Ethics statement

The study involved surveys with human participants. The ethics statement indicates approval by an Institutional Committee for the Care and Use of Animals in Research. The authors should clarify whether this committee had formal authority to review human-subject research involving surveys. If applicable, approval or exemption from an appropriate human research ethics committee should be reported.

7.Language and terminology

The manuscript is intelligible and written in generally understandable English. Nevertheless, it would benefit from some English language editing. Some expressions should be corrected or refined, for example “Emerging of infectious diseases” (should say emergence), “Whitin”, and “natural refugees”. The term “sanitary risk” should also be reconsidered in several places; depending on the context, “health risk”, “public health risk”, or “risk of exposure” may be more appropriate. Please also standardize terminology such as “Los Ríos”, “southern Chile”, “households”, “dwellings”, and “bat presence” throughout the manuscript.

Overall, this is a valuable descriptive contribution that provides relevant baseline evidence for understanding bat-human-domestic animal interfaces in rural southern Chile. After clarification of the derived variables, improvement of data availability, minor refinements in interpretation, and correction of figure and language issues, the manuscript would be suitable for publication.

**********

6. PLOS authors have the option to publish the peer review history of their article (what does this mean?). If published, this will include your full peer review and any attached files.

If you choose “no”, your identity will remain anonymous but your review may still be made public.

**Do you want your identity to be public for this peer review?**  For information about this choice, including consent withdrawal, please see our Privacy Policy.

Reviewer #1: **Yes:**  Aaron J. Enriquez

Reviewer #2: No

**********

[NOTE: If reviewer comments were submitted as an attachment file, they will be attached to this email and accessible via the submission site. Please log into your account, locate the manuscript record, and check for the action link "View Attachments". If this link does not appear, there are no attachment files.]

To ensure your figures meet our technical requirements, please review our figure guidelines: https://journals.plos.org/plosone/s/figures

You may also use PLOS’s free figure tool, NAAS, to help you prepare publication quality figures: https://journals.plos.org/plosone/s/figures#loc-tools-for-figure-preparation.

NAAS will assess whether your figures meet our technical requirements by comparing each figure against our figure specifications.

# Author comment: Author response to Decision Letter 1

**DOI:** 10.1371/journal.pone.0353070.r002

22 May 2026

Title: Perceptions and public health risks of the bat-human interface in households from fragmented rural landscapes in southern Chile

Dear Editor and Staff,

Thank you for the opportunity to revise our manuscript. We have carefully reviewed the PLOS ONE style requirements and have implemented the following changes to ensure full compliance with the journal's formatting guidelines:

1. Compliance with Style Requirements and File Naming

All headings, affiliations, citations and references have been adjusted to meet the journal's guidelines.

Figure 1 Copyright Compliance: We clarify that Figure 1 is an original map generated by the authors using R software. The administrative boundary data used as base layers (Comunas.geojson and Regional.geojson) were obtained from official Chilean government repositories, which are in the public domain. No proprietary data or software (e.g., Google Maps, Google Earth) were used in the creation of this figure.

Figure 2 Copyright Compliance: We clarify that Figure 2 consists of original photographs taken by the authors during the fieldwork activities for this study. The authors hold the copyright for these images and authorize their publication under the CC BY 4.0 license.

Figure 3 Copyright Compliance: We clarify that Figure 3 is an original illustration created by the authors. The silhouettes of the cat, elderly woman, infant, cow, and chicken are in the public domain (CC0 1.0). The dog silhouette (Canis familiaris dingo) is an icon by Sam Fraser-Smith (photo) and T. Michael Keesey (silhouette), which is available under a CC BY license. We have updated the figure legend to include the appropriate attribution as required by the license and PLOS ONE policies.

2. Data Availability Statement

We have updated our Data Availability Statement in the online submission form and the manuscript. Following the journal's policy, we have removed the co-author as a contact point and replaced it with a non-author institutional contact.

Revised Data Statement: "The data supporting the findings of this study are available upon reasonable request for researchers who meet the criteria for access to confidential data. Due to ethical and privacy restrictions regarding human participants in rural communities, data are not publicly available in a repository. Requests for data access can be directed to the Comité Institucional de Cuidado y Uso de Animales (CICUA-UACh) at the following institutional address:

Contact Name/Body: CICUA-UACh.

Email: comiteusoanimales@uach.cl

3. Funding Statement and Competing Interests

We thank the editor for bringing this to our attention. However, we would like to clarify that author Annia Rodríguez-San Pedro’s (AR-SP) affiliation was inadvertently listed as commercial in the previous version. Her participation in this study corresponds strictly to her academic affiliation. We have updated her affiliation in the revised manuscript and the online submission form to accurately reflect this: Centro de Investigación e Innovación para el Cambio Climático, Facultad de Ciencias, Universidad Santo Tomás, Santiago, Chile.

Consequently, Bioecos EIRL did not provide salary or any other financial support for this study, and there are no commercial competing interests to declare. We provide our updated statements below:

Updated Funding Statement: "This study was supported by the National Agency for Research and Development (ANID) through the ANILLO project ATE220062. The funders had no role in study design, data collection and analysis, decision to publish, or preparation of the manuscript."

Updated Competing Interests Statement: "The authors have declared that no competing interests exist”.

Reviewer 1:

1. The survey included a question asking people if they're vaccinated against rabies. I would have liked to see those responses summarized in the paper. Going a step further: it would be valuable to also show information on population rabies vaccination rates in the Los Ríos and Los Lagos regions. This could allow you to draw an interesting comparison of how the sample vaccination rates compare to the population vaccination rates.

Author´s response: We thank the reviewer for this valuable suggestion. We have now included the summarized responses regarding human rabies vaccination in the results section of the manuscript. Out of 69 surveyed households, only 13.0% (n=9) reported that they or a family member had been vaccinated against rabies, while 79.7% (n=55) reported not being vaccinated (the remaining 7.2% did not know or left the question blank).

Regarding the suggestion to compare our sample rates with population rabies vaccination rates in the Los Ríos and Los Lagos regions, it is important to clarify a key aspect of public health policy in Chile. Human rabies vaccination is not part of the universal routine immunization schedule. According to the Chilean Ministry of Health (MINSAL), human rabies vaccines are strictly administered as Post-Exposure Prophylaxis (PEP) for individuals bitten by suspect animals (such as dogs or wild mammals) or exposed to bats, and as pre-exposure prophylaxis for specific high-risk occupational groups (Ministerio de Salud, 2014).

Consequently, a "population vaccination rate" or coverage metric does not exist for humans in Chile as it does for routine vaccines. The 13% of respondents in our survey who reported being vaccinated likely represent individuals who received PEP at some point in their lives due to an animal bite or bat exposure. However, to provide the regional context the reviewer rightly points out, we have added official data to the discussion section regarding the incidence of PEP administration. In 2023, 2,453 first doses of the antirabies vaccine were administered in the Los Ríos region, and 4,702 first doses were administered in the Los Lagos region (Luchsinger et al.,2025). We believe this contextualizes the actual dynamics of rabies vaccination in these territories.

2. You mention that people have a low perception of the actual risk of rabies transmission to humans in the country. But, I was left wondering, what is the actual risk of rabies transmission? Again, could you provide statistics for rabies rates in the populations of the two regions considered in the paper? This would help a reader conceptualize how big of a risk rabies actually is.

Author´s response: We appreciate the reviewer’s request for clarification, as it highlights a crucial epidemiological paradox in our study area. To address this, it is important to state that the actual incidence rate of human rabies in Chile and specifically in the Los Ríos and Los Lagos regions is practically zero.

Chile was declared free of human rabies transmitted by dogs decades ago, with the last canine-variant case reported in 1972 (Ministerio de Salud, 2021; Luchsinger et al., 2025). Since the discovery of bats as the main reservoir in 1985, only two human rabies cases have been reported nationally: one in 1996 (from a bat variant) and another in 2013 (an encephalitis case without virological confirmation) (Luchsinger et al., 2025; Alegría-Moran et al., 2017). Neither of these cases occurred in the Los Ríos or Los Lagos regions.

Therefore, providing a statistical 'rabies rate' for the human populations in our studied regions would yield 0 cases per 100,000 inhabitants. This extremely low disease incidence is precisely the root of the problem: because people do not see or hear about human rabies cases, they do not perceive bats as a tangible threat. However, this zero human incidence occurs despite the virus actively circulating in the environment. Longitudinal data from the National Rabies Surveillance Program (Instituto de Salud Pública - ISP) shows that rabies positivity among tested bats nationwide consistently ranges between 7% and 10%, reaching 8.7% in 2023 and 8.6% in 2024. Furthermore, the virus is highly active in our specific study area; for instance, bats tested in the Los Lagos region recorded a 17.4% positivity rate in 2023 (ISP, 2023; 2024).

As our study shows, the exposure hazard is high due to frequent human-bat cohabitation, and this risk is actively managed by the health system through the high regional demand for Post-Exposure Prophylaxis (PEP) that we discussed in the previous comment. We have added a paragraph to the discussion to clarify the actual incidence of the disease, the sustained viral prevalence in local bat populations, and how this near-absence of human cases drives the low risk perception.

3. You mentioned that dwellings/structures in the immediate vicinity were visited. I'm wondering, was there any specific threshold distance beyond which you did not consider dwellings/structures as eligible? Also, was there a target or maximum number of dwellings/structures you targeted per each sampling point?

Author´s response: We thank the reviewer for this question, which allows us to clarify our sampling approach at the household level. We did not establish a specific threshold distance in meters, nor a maximum number of targeted structures per sampling point. Instead, our operational boundary was the fenced perimeter of each selected property.

As it is common in rural areas of southern Chile for a single property to contain more than one building (e.g., a main house, a secondary house for extended family, sheds, or even small community structures like a local church where the homeowner is the pastor), all structures within the property limits were considered eligible and visited. We have updated the Methods section in the revised manuscript to clearly reflect this operational definition.

4. You have a sentence about excluding surveys if fewer than 75% of questions were answered or if critical missing information could not be resolved after three attempted contacts. Please provide readers with the specific number of surveys that fell into those categories and thus were omitted.

Author´s response: We thank the reviewer for pointing out this omission. We agree that providing the exact number of excluded surveys provides greater transparency to our data collection process. As requested, we have updated the manuscript to explicitly state that a total of 72 surveys were initially obtained, of which 3 did not meet the data quality criteria and were therefore omitted, leaving 69 correctly completed surveys for the final analysis.

5. You settled on using three contact levels (low, medium, high). That makes sense, but it would be great if you provided readers a little more information on how exactly you settled on using these three levels, as well as what sorts of specific rates constitute things like "high frequency access" to bats.

Author´s response: We thank the reviewer for this constructive comment. We agree that the rationale behind our contact level categorization needed more clarity for the readers. The three levels (low, medium, high) were established to reflect a gradient of epidemiological risk, moving from mere environmental overlap (lowest risk) to potential indoor exposure (medium risk), and finally to direct physical exposure or unavoidable proximity (highest risk, direct contact).

Regarding "high frequency access", since our cross-sectional survey did not measure a specific numerical rate (e.g., number of interactions per week), we realize the phrasing could be misinterpreted. We used this term to describe qualitative scenarios where the spatial overlap makes recurrent encounters practically unavoidable, for instance, when a bat colony is roosting directly inside high-traffic living areas (such as bedrooms or kitchens), or when residents or pets repeatedly interact with bats while trying to remove them from the living space. We have expanded the methodology section to explicitly detail what constitutes each level with concrete examples, and we have clarified the definition of “high frequency access”.

6. The action category variable includes three groups: negative/aggressive, neutral/evasive, and precautionary/informative. However, I'm concerned that the breakdown may not be clean. For example, what if someone is the type of person that engages in preventative measures (thus falling into the precautionary/informative group), but then a bat makes its way into their house anyway, and then they react in a negative/aggressive way (thus also falling into the negative/aggressive category)? This would mean that the categories aren't mutually exclusive.

Author´s response: We thank the reviewer for this perceptive comment. We realize that our use of the phrase 'preventive measures' when defining the “precautionary/informative” category caused unintended confusion. To clarify, this variable was not designed to classify a respondent's general lifestyle or prior structural prevention habits (e.g., sealing gaps in the house). Instead, it exclusively measures the respondent's primary, immediate reaction during the specific event of finding a bat indoors.

Because the open-ended responses focused solely on the immediate action taken during the encounter, the categories are mutually exclusive. Each of the 18 respondents described a primary course of action for that specific moment, allowing them to be classified into an unique group. To prevent the exact confusion the reviewer correctly pointed out, we have rephrased the definition of the “precautionary/informative” category in the methodology, replacing “preventive measures” with “safe containment or precautionary actions during the encounter”. We believe this clearly separates the immediate reaction to an indoor bat from general preventative household maintenance.

7. In Figure 1, given that you use color, I would recommend making water appear a light blue in the map.

Author´s response: We thank the reviewer for this recommendation. We agree that this change improves the visual clarity of the map. Figure 1 has been updated accordingly, and the water is now displayed in light blue.

8. You provide summary statistics for people with dogs and people with cats. I'm wondering whether it might be interesting to distinguish this a little more specifically: people with dogs only, people with cats only, and people with both dogs and cats.

Author´s response: We thank the reviewer for this suggestion. We agree that breaking down pet ownership into mutually exclusive categories provides a much clearer and interesting picture of the domestic animal interface in our study area.

Based on our data, out of the 69 surveyed households, 44.9% (n=31) owned both dogs and cats, 20.3% (n=14) owned only dogs, and 5.8% (n=4) owned only cats, accounting for the 49 households that reported having pets. We have updated the 'Household sample and baseline characteristics' subsection in the Results to include this detailed and specific breakdown, replacing the previous summary statistics.

Reviewer 2:

1. Clarification of the derived contact-level variable

The ordinal “human-bat contact level” variable is central to the analysis and interpretation of the results. However, the manuscript should describe more explicitly how this variable was constructed from the questionnaire responses. I suggest adding a supplementary coding table indicating which specific answers were used to classify households as low, medium, or high contact. This would improve reproducibility and allow readers to better understand the analytical approach.

Author´s response: We thank the reviewer for this constructive suggestion, which indeed enhances the transparency and reproducibility of our study. In response to a related comment raised by Reviewer 1 regarding the definition of these categories, we have already expanded the text in the Methods section to explicitly detail the epidemiological risk gradient defining the “low”, “medium”, and “high” contact levels, and clarified qualitative terms such as “high frequency access”.

Furthermore, we completely agree with your suggestion to provide a coding matrix. We have now created and referenced a new supplementary table (S2 Table) that explicitly maps the specific questionnaire responses and reported scenarios used to classify each household into its respective contact level. We believe this addition provides the exact clarity requested.

2. Clarification of the knowledge score

The health-risk know

Attachment Submitted filename: Response to Editorial Requirements_CV.docx

# Peer review: Decision Letter 1

**DOI:** 10.1371/journal.pone.0353070.r003

**Authors:** Daniel Oluwayelu, Daniel Oluwayelu

10 Jun 2026

PONE-D-26-17161R1

Perceptions and public health risks of the bat-human interface in households from fragmented rural landscapes in southern Chile.

PLOS One

Dear Dr. Hernandez,

Thank you for submitting your manuscript to PLOS ONE. After careful consideration, we feel that it has merit but does not fully meet PLOS ONE’s publication criteria as it currently stands. Therefore, we invite you to submit a revised version of the manuscript that addresses the points raised during the review process, especially the minor and very minor comments of Reviewer 1.

Please submit your revised manuscript by Jul 25 2026 11:59PM. If you will need more time than this to complete your revisions, please reply to this message or contact the journal office at plosone@plos.org. When you're ready to submit your revision, log on to https://www.editorialmanager.com/pone/ and select the 'Submissions Needing Revision' folder to locate your manuscript file.

Please include the following items when submitting your revised manuscript:

- A letter that responds to each point raised by the academic editor and reviewer(s). You should upload this letter as a separate file labeled 'Response to Reviewers'.
- A marked-up copy of your manuscript that highlights changes made to the original version. You should upload this as a separate file labeled 'Revised Manuscript with Track Changes'.
- An unmarked version of your revised paper without tracked changes. You should upload this as a separate file labeled 'Manuscript'.

If you would like to make changes to your financial disclosure, please include your updated statement in your cover letter. Guidelines for resubmitting your figure files are available below the reviewer comments at the end of this letter.

If applicable, we recommend that you deposit your laboratory protocols in protocols.io to enhance the reproducibility of your results. Protocols.io assigns your protocol its own identifier (DOI) so that it can be cited independently in the future. For instructions see: https://journals.plos.org/plosone/s/submission-guidelines#loc-laboratory-protocols. Additionally, PLOS ONE offers an option for publishing peer-reviewed Lab Protocol articles, which describe protocols hosted on protocols.io. Read more information on sharing protocols at https://plos.org/protocols?utm_medium=editorial-email&utm_source=authorletters&utm_campaign=protocols.

As the corresponding author, your ORCID iD is verified in the submission system and will appear in the published article. PLOS supports the use of ORCID, and we encourage all coauthors to register for an ORCID iD and use it as well. Please encourage your coauthors to verify their ORCID iD within the submission system before final acceptance, as unverified ORCID iDs will not appear in the published article. *Only*  the individual author can complete the verification step; PLOS staff *cannot*  verify ORCID iDs on behalf of authors.

We look forward to receiving your revised manuscript.

Kind regards,

Daniel Oladimeji Oluwayelu, D.V.M., M.Sc., Ph.D.

Academic Editor

PLOS One

Journal Requirements:

If the reviewer comments include a recommendation to cite specific previously published works, please review and evaluate these publications to determine whether they are relevant and should be cited. There is no requirement to cite these works unless the editor has indicated otherwise.

Please review your reference list to ensure that it is complete and correct. If you have cited papers that have been retracted, please include the rationale for doing so in the manuscript text, or remove these references and replace them with relevant current references. Any changes to the reference list should be mentioned in the rebuttal letter that accompanies your revised manuscript. If you need to cite a retracted article, indicate the article’s retracted status in the References list and also include a citation and full reference for the retraction notice.

[Note: HTML markup is below. Please do not edit.]

Reviewers' comments:

Reviewer's Responses to Questions

**Comments to the Author**

1. If the authors have adequately addressed your comments raised in a previous round of review and you feel that this manuscript is now acceptable for publication, you may indicate that here to bypass the “Comments to the Author” section, enter your conflict of interest statement in the “Confidential to Editor” section, and submit your "Accept" recommendation.

Reviewer #1: (No Response)

Reviewer #2: All comments have been addressed

**********

2. Is the manuscript technically sound, and do the data support the conclusions?

The manuscript must describe a technically sound piece of scientific research with data that supports the conclusions. Experiments must have been conducted rigorously, with appropriate controls, replication, and sample sizes. The conclusions must be drawn appropriately based on the data presented.

Reviewer #1: Yes

Reviewer #2: Yes

**********

3. Has the statistical analysis been performed appropriately and rigorously?

Reviewer #1: Yes

Reviewer #2: Yes

**********

4. Have the authors made all data underlying the findings in their manuscript fully available?

The PLOS Data policy requires authors to make all data underlying the findings described in their manuscript fully available without restriction, with rare exception (please refer to the Data Availability Statement in the manuscript PDF file). The data should be provided as part of the manuscript or its supporting information, or deposited to a public repository. For example, in addition to summary statistics, the data points behind means, medians and variance measures should be available. If there are restrictions on publicly sharing data—e.g. participant privacy or use of data from a third party—those must be specified.

Reviewer #1: No

Reviewer #2: Yes

**********

5. Is the manuscript presented in an intelligible fashion and written in standard English?

PLOS ONE does not copyedit accepted manuscripts, so the language in submitted articles must be clear, correct, and unambiguous. Any typographical or grammatical errors should be corrected at revision, so please note any specific errors here.

Reviewer #1: Yes

Reviewer #2: Yes

**********

6. Review Comments to the Author

Please use the space provided to explain your answers to the questions above. You may also include additional comments for the author, including concerns about dual publication, research ethics, or publication ethics. (Please upload your review as an attachment if it exceeds 20,000 characters)

Reviewer #1: Job well done to the authors, who satisfactorily addressed all of my prior comments. I believe this manuscript is in great shape, and I have no more major comments. However, I have a few additional minor and very minor comments from this latest version of the manuscript and supplemental files. I will list those below, hopefully the authors find them helpful.

Minor comments

You use the term "exposure opportunities" a few times throughout the manuscript. Maybe it's just me, but I associate the word "opportunity" in somewhat of a positive light. Would recommend considering an alternative word instead.

Line 255: Prior to this paragraph of the Results section, you were typically listing fractions first, then percentages, but it swaps here. Also, sometimes you use a fraction for the numbers, whereas other times you use "n=". Recommend being consistent with all these types of things across the entire section.

I'm liking the additions to this Discussion section. However, currently, there is a very long block of text to end the section. Would recommend splitting out paragraphs here:

- Line 431: Make the "This lack of…" a new paragraph

- Line 447: Make the "Several limitations should…" a new paragraph

Very minor comments

Line 17: In footnote 7, wondering if "climático" should be capitalized.

Line 26: Appears that there could be more than one space between "health" and "risk"

Line 30: Would sound better as "with 67% of dwellings presenting"

Line 60: You use "refugees" here

Line 64: Consider hyphenating "highly urbanized"

Line 71: Would recommend replacing "coexists" with "coincides"

Lines: 76-77: The "although still is an" part reads unnaturally. Would suggest a rewrite.

Line 79: Here you use "Rios" instead of "Ríos"

Line 137: Would recommend moving the "(bat feces)" part right before the comma in the next line

Line 139: There may be an additional space between the comma and "use"?

Line 174: Consider hyphenating "mutually exclusive"

Line 180: Here, you use a greater than or equal to sign before 65 years. Elsewhere (e.g., line 134) you use just a greater than sign. Please pick one and be consistent throughout.

Line 182: Recommend no comma

Line 200: Consider using "bat" and "rodent" singular here, it may sound better

Line 205: "under the fact" is an odd term, recommend rewording

Line 221: Need a space between the period after the parentheses and "Surveyed"

Line 221: Add an "a" such that it reads "across a diverse range"

Table 2: The left column appears top-aligned while the subsequent columns appear middle- or bottom-aligned. Would recommend making them all have top-alignment.

Line 292: You mention "S3-S7 Table". Consider using "Tables S3-S7" instead. If so, please update this also in line 635 and within the corresponding supplement document.

Line 335: Remove the "s" from "domestic animals"

Line 340: "are all" may sound better

Line 360: add "bats" so that it's "bringing bats into"

Line 370: Rewrite (e.g., "In children, increased curiosity or lower perception of danger could…")

Line 373: In both this sentence and the one before it, you use "or" before the last term in your lists. Would recommend "and" instead.

Lines 374-375: Need commas around "which supports their role in facilitating bat entry"

Line 379: Here you use "Rios" instead of "Ríos"

Line 403: Becomes a little unclear in this sentence what the intended subject is, whether it's the rabies, the spillovers, or both. If it's both, should use "are" instead of "is"

Line 412: Use "disconnect" instead of "disconnection"

Line 470: Remove the unnecessary ""."

S1 Table document: would top-align the left-most column in the table to make the rows a little easier to follow.

S2 Table document: Would make the "Lowest", "Moderate", and "Highest" terms in parentheses (in column 2) be lower-case.

S2 Table document: In the final row and column of the table, are you missing an "OR" before the last sentence?

S3-S7 Table document: Some terms throughout this document seem randomly capitalized (e.g., "Dog presence", "Human-bat contact", "Vulnerable Population", etc.). Would recommend making them all lower-case.

Reviewer #2: The authors have satisfactorily addressed my main comments and have substantially improved the manuscript. In particular, the revised version provides a clearer description of the sampling approach, the construction of the ordinal contact-level variable, the exploratory nature of the statistical analyses, the interpretation of public health risk as exposure opportunities rather than direct measurement of pathogen transmission, and the ethical approval process for the household survey.

I recommend acceptance

**********

7. PLOS authors have the option to publish the peer review history of their article (what does this mean?). If published, this will include your full peer review and any attached files.

If you choose “no”, your identity will remain anonymous but your review may still be made public.

**Do you want your identity to be public for this peer review?**  For information about this choice, including consent withdrawal, please see our Privacy Policy.

Reviewer #1: **Yes:**  Aaron J. Enriquez

Reviewer #2: No

**********

[NOTE: If reviewer comments were submitted as an attachment file, they will be attached to this email and accessible via the submission site. Please log into your account, locate the manuscript record, and check for the action link "View Attachments". If this link does not appear, there are no attachment files.]

To ensure your figures meet our technical requirements, please review our figure guidelines: https://journals.plos.org/plosone/s/figures

You may also use PLOS’s free figure tool, NAAS, to help you prepare publication quality figures: https://journals.plos.org/plosone/s/figures#loc-tools-for-figure-preparation.

NAAS will assess whether your figures meet our technical requirements by comparing each figure against our figure specifications.

# Author comment: Author response to Decision Letter 2

**DOI:** 10.1371/journal.pone.0353070.r004

15 Jun 2026

Manuscript ID:

Title: Perceptions and public health risks of the bat-human interface in households from fragmented rural landscapes in southern Chile

RESPONSE TO REVIEWER 1

General Comment:

Job well done to the authors, who satisfactorily addressed all of my prior comments. I believe this manuscript is in great shape, and I have no more major comments. However, I have a few additional minor and very minor comments from this latest version of the manuscript and supplemental files. I will list those below, hopefully the authors find them helpful.

Response: We sincerely thank the reviewer for their positive assessment, encouraging words, and dedication to refining our manuscript across these revision rounds. We have addressed each of the remaining minor and very minor comments to ensure complete clarity and consistency

Minor comments

Comment 1: You use the term "exposure opportunities" a few times throughout the manuscript. Maybe it's just me, but I associate the word "opportunity" in somewhat of a positive light. Would recommend considering an alternative word instead.

Response: We appreciate this perceptive semantic observation. We agree that the term "opportunity" can carry an unintended positive connotation that is counterintuitive within a public health context. To address this, we have systematically reviewed the manuscript and replaced "exposure opportunities" with more precise, context-dependent epidemiological terms (such as "potential for exposure", "exposure pathways", "exposure risk", or "likelihood of exposure").

Comment 2: Line 255: Prior to this paragraph of the Results section, you were typically listing fractions first, then percentages, but it swaps here. Also, sometimes you use a fraction for the numbers, whereas other times you use "n=". Recommend being consistent with all these types of things across the entire section.

Response: We thank the reviewer for their meticulous reading and for pointing out these stylistic inconsistencies. We have thoroughly audited the entire Results section to ensure data reporting uniformity.

Comment 3: I'm liking the additions to this Discussion section. However, currently, there is a very long block of text to end the section. Would recommend splitting out paragraphs here: - Line 431: Make the "This lack of…" a new paragraph - Line 447: Make the "Several limitations should…" a new paragraph.

Response: We completely agree that breaking up this long block of text significantly enhances the readability and structural flow of our final arguments. Accordingly, we have restructured this section by creating new paragraphs at the exact locations requested.

Very Minor Comments

Comment 4: Line 17: In footnote 7, wondering if "climático" should be capitalized.

Response: We thank the reviewer for catching this typographical error. "Climático" has now been properly capitalized, as it is part of the formal name of the research center (Centro de Investigación e Innovación para el Cambio Climático).

Comment 5: Line 26: Appears that there could be more than one space between "health" and "risk"

Response: The text has been corrected, and the extra space between "health" and "risk" has been removed.

Comment 6: Line 30: Would sound better as "with 67% of dwellings presenting"

Response: We agree with the reviewer's stylistic suggestion to improve the sentence flow. The text in the Abstract has been updated accordingly.

Comment 7: Line 60: You use "refugees" here

Response: We thank the reviewer for catching this typographical error. We have corrected the word "refugees" to "refuges" to accurately refer to bat roosts and shelters.

Comment 8: Line 64: Consider hyphenating "highly urbanized"

Response: Done. The phrase has been hyphenated to "highly-urbanized" to improve grammatical accuracy.

Comment 9: Line 71: Would recommend replacing "coexists" with "coincides"

Response: Done. We have replaced "coexists" with "coincides" to better reflect the spatial-temporal alignment described in that section.

Comment 10: Lines: 76-77: The "although still is an" part reads unnaturally. Would suggest a rewrite.

Response: We have rewritten this clause to improve clarity and sentence flow. The sentence now reads as follows: "...representing a critical component in understanding zoonotic risk, although it remains an insufficiently characterized dimension of spillover prevention."

Comment 11: Line 79: Here you use "Rios" instead of "Ríos"

Response: We thank the reviewer for their careful proofreading. The missing accent mark has been added, and the text has been corrected to "Ríos".

Comment 12: Line 137: Would recommend moving the "(bat feces)" part right before the comma in the next line

Response: Done. We have relocated the parenthetical term "(bat feces)" right before the comma on the following line to improve sentence structure and readability.

Comment 13: Line 139: There may be an additional space between the comma and "use"?

Response: Done. The text has been corrected, and the additional space after the comma has been removed.

Comment 14: Line 174: Consider hyphenating "mutually exclusive"

Response: Done. The phrase has been hyphenated to "mutually-exclusive" to ensure stylistic and grammatical consistency.

Comment 15: Line 180: Here, you use a greater than or equal to sign before 65 years. Elsewhere (e.g., line 134) you use just a greater than sign. Please pick one and be consistent throughout.

Response: We thank the reviewer for noting this inconsistency. We have standardized this threshold across the entire manuscript by using the greater than or equal to sign (≥) before 65 years (i.e., ≥65 years), as this precisely aligns with our demographic categorization and survey design for the senior cohort (65 years or older). Line 134 and all other relevant instances have been corrected to ensure full text uniformity.

Comment 16: Line 182: Recommend no comma

Response: Done. The unnecessary comma has been removed from this sentence.

Comment 17: Line 200: Consider using "bat" and "rodent" singular here, it may sound better

Response: Done. We have changed both terms to their singular forms ("bat" and "rodent") as suggested, which significantly improves the phrasing of this sentence.

Comment 18: Line 205: "under the fact" is an odd term, recommend rewording

Response: Done. We agree that this phrasing was awkward. We have rephrased the sentence to use "given that" instead. It now reads: "...and were specifically reviewed and approved by the aforementioned committee, given that some of the animal traps were placed within the interviewees' properties."

Comment 19: Line 221: Need a space between the period after the parentheses and "Surveyed"

Response: Done. The missing space has been inserted between the period and the word "Surveyed" to correct this formatting oversight.

Comment 20: Line 221: Add an "a" such that it reads "across a diverse range"

Response: Done. We have added the missing article "a" so that the text now properly reads "across a diverse range".

Comment 21: Table 2: The left column appears top-aligned while the subsequent columns appear middle- or bottom-aligned. Would recommend making them all have top-alignment

Response: Done. We have modified the structural formatting of Table 2 so that all cells across all columns are now uniformly top-aligned.

Comment 22: Line 292: You mention "S3-S7 Table". Consider using "Tables S3-S7" instead. If so, please update this also in line 635 and within the corresponding supplement document.

Response: Done. We thank the reviewer for this formatting check to align our text with standard journal style. We have updated the reference to "Tables S3–S7" in both line 292 and line 635 of the revised manuscript.

Comment 23: Line 335: Remove the "s" from "domestic animals"

Response: Done. We have removed the plural "s" to change the phrase to "domestic animal" as recommended, ensuring proper grammatical agreement within that sentence

Comment 24: Line 340: "are all" may sound better

Response: Done. We have adjusted the phrasing to "They are all insectivorous..." as recommended, which significantly improves the linguistic flow of this sentence.

Comment 25: Line 360: add "bats" so that it's "bringing bats into"

Response: Done. We have added the word "bats" to the text, so the phrase now reads "bringing bats into" exactly as suggested to ensure full clarity

Comment 26: Line 370: Rewrite (e.g., "In children, increased curiosity or lower perception of danger could…")

Response: Done.

The sentence has been rewritten as follows: "In children, increased curiosity or lower perception of danger could increase the likelihood of approaching or handling bats."

Comment 27: Lines 374-375: Need commas around "which supports their role in facilitating bat entry"

Response: Done.

The sentence now reads as follows: "...In contrast, although structural openings were highly prevalent, which supports their role in facilitating bat entry, they were not significantly associated with higher levels of human-bat contact."

Comment 28: Line 379: Here you use "Rios" instead of "Ríos"

Response: Done. The missing accent mark has been added, and the text has been corrected to "Ríos" to ensure spelling accuracy and consistency across the entire manuscript.

Comment 29: Line 403: Becomes a little unclear in this sentence what the intended subject is, whether it's the rabies, the spillovers, or both. If it's both, should use "are" instead of "is"

Response: Done. We thank the reviewer for pointing out this ambiguity. The intended subject is indeed a single, integrated concept: the spillover of rabies. To make this explicitly clear and resolve the confusion caused by the proximity of the plural word "spillovers", we have rephrased the subject to the singular "Bat-borne rabies spillover". We have also corrected "veterinarian issue" to "veterinary issue" for grammatical precision.

The sentence has been rewritten as follows: "Bat-borne rabies spillover to livestock is primarily documented as a veterinary issue, where surveillance data confirms bats as the primary reservoir maintaining viral variants that spill over into productive herds through accidental encounters”

Comment 30: Line 412: Use "disconnect" instead of "disconnection"

Response: Done. We have replaced "disconnection" with "disconnect" as suggested, which improves the phrasing and overall flow of the sentence.

Comment 31: Line 470: Remove the unnecessary ""."

Response: Done.

Comment 32: S1 Table document: would top-align the left-most column in the table to make the rows a little easier to follow.

Response: Done. We have adjusted the vertical alignment of the leftmost column in the S1 Table to be top-aligned.

Comment 33: S2 Table document: Would make the "Lowest", "Moderate", and "Highest" terms in parentheses (in column 2) be lower-case.

Response: Done. We have updated column 2 of the S2 Table document so that the terms in parentheses ("lowest", "moderate", and "highest") are now lowercase, ensuring consistent and clean formatting throughout the table.

Comment 34: S2 Table document: In the final row and column of the table, are you missing an "OR" before the last sentence?

Response: Done. We thank the reviewer for pointing out this omission.

The text in that cell has been updated as follows: "“Yes” to: “Have you observed contact between bats and your domestic animals (pets)?” OR “Have you observed contact between bats and your production animals?” OR Qualitative open-ended reports derived from: “Have you taken control measures to remove them?” (e.g., direct handling, capturing, or killing the bat) OR “Please characterize the structure housing the bat colony” (e.g., roosts located directly inside high-traffic living areas like bedrooms or kitchens, defining high frequency access)."

Comment 35: S3-S7 Table document: Some terms throughout this document seem randomly capitalized (e.g., "Dog presence", "Human-bat contact", "Vulnerable Population", etc.). Would recommend making them all lower-case.

Response: Done. We thank the reviewer for noting these capitalization inconsistencies. We have thoroughly reviewed the supplementary document containing Tables S3–S7 and converted all randomly capitalized terms within the tables to lowercase (e.g., changing them to "dog presence", "human-bat contact", and "vulnerable population"), ensuring a clean and uniform formatting standard throughout the files.

RESPONSE TO REVIEWER 2

General Comment:

The authors have satisfactorily addressed my main comments and have substantially improved the manuscript. In particular, the revised version provides a clearer description of the sampling approach, the construction of the ordinal contact-level variable, the exploratory nature of the statistical analyses, the interpretation of public health risk as exposure opportunities rather than direct measurement of pathogen transmission, and the ethical approval process for the household survey. I recommend acceptance.

Response: We are grateful to the reviewer for their positive evaluation and for recommending the acceptance of our manuscript. We appreciate the time, expertise, and constructive feedback they provided during the previous revision rounds.

Attachment Submitted filename: Response_to_Editorial_Requirements_CV_auresp_2.docx

# Peer review: Decision Letter 2

**DOI:** 10.1371/journal.pone.0353070.r005

**Authors:** Daniel Oluwayelu, Daniel Oluwayelu, Daniel Oluwayelu

18 Jun 2026

Perceptions and public health risks of the bat-human interface in households from fragmented rural landscapes in southern Chile.

PONE-D-26-17161R2

Dear Dr. Hernandez,

We’re pleased to inform you that your manuscript has been judged scientifically suitable for publication and will be formally accepted for publication once it meets all outstanding technical requirements.

Within one week, you’ll receive an e-mail detailing the required amendments. When these have been addressed, you’ll receive a formal acceptance letter and your manuscript will be scheduled for publication.

An invoice will be generated when your article is formally accepted. Please note, if your institution has a publishing partnership with PLOS and your article meets the relevant criteria, all or part of your publication costs will be covered. Please make sure your user information is up-to-date by logging into Editorial Manager at Editorial Manager® and clicking the ‘Update My Information' link at the top of the page. For questions related to billing, please contact billing support.

If your institution or institutions have a press office, please notify them about your upcoming paper to help maximize its impact. If they’ll be preparing press materials, please inform our press team as soon as possible -- no later than 48 hours after receiving the formal acceptance. Your manuscript will remain under strict press embargo until 2 pm Eastern Time on the date of publication. For more information, please contact onepress@plos.org.

Kind regards,

Daniel Oladimeji Oluwayelu, D.V.M., M.Sc., Ph.D.

Academic Editor

PLOS One

Additional Editor Comments (optional):

Reviewers' comments:

Reviewer's Responses to Questions

**Comments to the Author**

1. If the authors have adequately addressed your comments raised in a previous round of review and you feel that this manuscript is now acceptable for publication, you may indicate that here to bypass the “Comments to the Author” section, enter your conflict of interest statement in the “Confidential to Editor” section, and submit your "Accept" recommendation.

Reviewer #1: All comments have been addressed

**********

2. Is the manuscript technically sound, and do the data support the conclusions?

The manuscript must describe a technically sound piece of scientific research with data that supports the conclusions. Experiments must have been conducted rigorously, with appropriate controls, replication, and sample sizes. The conclusions must be drawn appropriately based on the data presented.

Reviewer #1: Yes

**********

3. Has the statistical analysis been performed appropriately and rigorously?

Reviewer #1: Yes

**********

4. Have the authors made all data underlying the findings in their manuscript fully available?

The PLOS Data policy requires authors to make all data underlying the findings described in their manuscript fully available without restriction, with rare exception (please refer to the Data Availability Statement in the manuscript PDF file). The data should be provided as part of the manuscript or its supporting information, or deposited to a public repository. For example, in addition to summary statistics, the data points behind means, medians and variance measures should be available. If there are restrictions on publicly sharing data—e.g. participant privacy or use of data from a third party—those must be specified.

Reviewer #1: Yes

**********

5. Is the manuscript presented in an intelligible fashion and written in standard English?

PLOS ONE does not copyedit accepted manuscripts, so the language in submitted articles must be clear, correct, and unambiguous. Any typographical or grammatical errors should be corrected at revision, so please note any specific errors here.

Reviewer #1: Yes

**********

6. Review Comments to the Author

Please use the space provided to explain your answers to the questions above. You may also include additional comments for the author, including concerns about dual publication, research ethics, or publication ethics. (Please upload your review as an attachment if it exceeds 20,000 characters)

Reviewer #1: Fantastic work by the authors! They did a great job addressing all comments satisfactorily. This will make a nice contribution to the literature.

**********

7. PLOS authors have the option to publish the peer review history of their article (what does this mean?). If published, this will include your full peer review and any attached files.

If you choose “no”, your identity will remain anonymous but your review may still be made public.

**Do you want your identity to be public for this peer review?**  For information about this choice, including consent withdrawal, please see our Privacy Policy.

Reviewer #1: **Yes:**  Aaron J. Enriquez

**********

# Editor report: Acceptance letter

**DOI:** 10.1371/journal.pone.0353070.r006

**Authors:** Daniel Oluwayelu, Daniel Oluwayelu, Daniel Oluwayelu

PONE-D-26-17161R2

PLOS One

Dear Dr. Hernández-Agudelo,

I'm pleased to inform you that your manuscript has been deemed suitable for publication in PLOS One. Congratulations! Your manuscript is now being handed over to our production team.

At this stage, our production department will prepare your paper for publication. This includes ensuring the following:

* All references, tables, and figures are properly cited

* All relevant supporting information is included in the manuscript submission,

* There are no issues that prevent the paper from being properly typeset

You will receive further instructions from the production team, including instructions on how to review your proof when it is ready. Please keep in mind that we are working through a large volume of accepted articles, so please give us a few days to review your paper and let you know the next and final steps.

Lastly, if your institution or institutions have a press office, please let them know about your upcoming paper now to help maximize its impact. If they'll be preparing press materials, please inform our press team within the next 48 hours. Your manuscript will remain under strict press embargo until 2 pm Eastern Time on the date of publication. For more information, please contact onepress@plos.org.

You will receive an invoice from PLOS for your publication fee after your manuscript has reached the completed accept phase. If you receive an email requesting payment before acceptance or for any other service, this may be a phishing scheme. Learn how to identify phishing emails and protect your accounts at https://explore.plos.org/phishing.

If we can help with anything else, please email us at customercare@plos.org.

Thank you for submitting your work to PLOS ONE and supporting open access.

Kind regards,

PLOS ONE Editorial Office Staff

on behalf of

Professor Daniel Oladimeji Oluwayelu

Academic Editor

PLOS One
